# The Evolution of the Exponent of Zipf's Law in Language Ontogeny

**DOI:** 10.1371/journal.pone.0053227

**Published:** 2013-03-13

**Authors:** Jaume Baixeries, Brita Elvevåg, Ramon Ferrer-i-Cancho

**Affiliations:** 1 Laboratory for Relational Algorithmics, Complexity and Learning (LARCA), Departament de Llenguatges i Sistemes Informàtics, Universitat Politècnica de Catalunya, Barcelona, Catalonia, Spain; 2 Complexity & Quantitative Linguistics Lab, Departament de Llenguatges i Sistemes Informàtics, Center for Language and Speech Technologies and Applications (TALP Research Center), Universitat Politècnica de Catalunya, Barcelona, Catalonia, Spain; 3 Psychiatry Research Group, Department of Clinical Medicine, University of Tromsø, Tromsø, Norway; 4 Norwegian Centre for Integrated Care and Telemedicine (NST), University Hospital of North Norway, Tromsø, Norway; Wake Forest School of Medicine, United States of America

## Abstract

It is well-known that word frequencies arrange themselves according to Zipf's law. However, little is known about the dependency of the parameters of the law and the complexity of a communication system. Many models of the evolution of language assume that the exponent of the law remains constant as the complexity of a communication systems increases. Using longitudinal studies of child language, we analysed the word rank distribution for the speech of children and adults participating in conversations. The adults typically included family members (e.g., parents) or the investigators conducting the research. Our analysis of the evolution of Zipf's law yields two main unexpected results. First, in children the exponent of the law tends to decrease over time while this tendency is weaker in adults, thus suggesting this is not a mere mirror effect of adult speech. Second, although the exponent of the law is more stable in adults, their exponents fall below 1 which is the typical value of the exponent assumed in both children and adults. Our analysis also shows a tendency of the mean length of utterances (MLU), a simple estimate of syntactic complexity, to increase as the exponent decreases. The parallel evolution of the exponent and a simple indicator of syntactic complexity (MLU) supports the hypothesis that the exponent of Zipf's law and linguistic complexity are inter-related. The assumption that Zipf's law for word ranks is a power-law with a constant exponent of one in both adults and children needs to be revised.

## Introduction

Word frequencies arrange themselves according to Zipf's law [1], [2]. In his seminal work, G. K. Zipf showed that if the most frequent word in a text is assigned rank 1, the second most frequent word is assigned rank 2, and so on, then 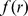,the frequency of a word of rank 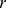 obeys [1]

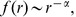
(1) where 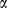 is the exponent of the law. 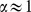 has been reported (e.g., [1]) or assumed (e.g., [3], [4]). From a mathematical perspective, Zipf's law can be formalized using a right-truncated zeta distribution [5]. Consider that ranks go from 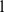 to a certain maximum value 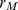. Then 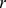 is distributed according to a right-truncated zeta distribution if and only if the probability of a word of rank 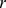 is [5]

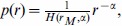
(2) where 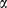 and 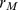 are the only parameters and 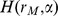, defined as
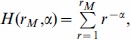
(3) is the generalized harmonic number of order 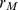 of 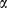. When 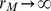 and 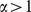, 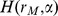 becomes 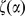, the Riemann zeta function, while 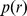 defines the zeta distribution [5] whose only parameter is 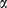.

A right-truncated zeta distribution for word ranks with 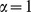 has been adopted in many models of the evolution of language [3], [6]–[8]. In particular, the models in [3], [7] assume that the exponent 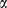 does not depend on whether a communication system has a rudimentary form of syntax or not while the model presented in [8] assumes that 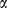 does not depend on a child's age or more importantly on key aspects of a child's language complexity such as the mean length of an utterance (MLU) in words (see [9], pp. 255, for an approximate time line of MLU's as a function of childrens' age). In contrast, certain theoretical models based upon Zipf's law for word frequencies have shown that various aspects of the complexity of a communication system (e.g., its capacity to combine words to build complex sentences) may depend on the value of the exponent [10], [11]. Values of 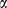 that clearly exceed 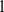 have been reported for children [12], [13] but a precise study of how the exponent evolves over time is lacking. In their pioneering work, McCowan and collaborators studied the development of communication through Zipf's law in humans, dolphins (*Tursiops truncatus*) and arboreal squirrel monkeys (*Saimiri sciureus*) [14], and a bell-shaped evolution of the exponent of Zipf's law over time was suggested. Note that our conventions are different: while McCowan *et al*. treated the negative sign as part of the exponent [14] and thus suggested an inverted bell-shape for the relationship between their exponent and time, when following our notation 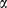 does not include it and thus translates into a bell-shape. However, McCowan *et al*. did not study actual age and their analysis was based on only a few groups of different ages (their analysis in humans was based on only two groups, namely, infants and adults). Thus, studying the evolution of the actual value of the exponent of Zipf's law as children get older and increase the complexity of their communication system is clearly needed.

Here we aim to shed light on the evolution of the exponent of Zipf's law in language ontogeny and go beyond the limits of previous approaches:

- Instead of only a few age categories [14] as many age points as possible are used.
- The speech of adults interacting with children is employed as a control, a methodological concern that is missing in [8].
- Instead of only a single language and only two children (as in [8]) we examined four languages and included over seventy children.
- The exponent of the law is obtained by maximum likelihood [15] to minimize estimation biases [16].
- Instead of estimating word frequency from parental language diaries or vocabulary check lists (e.g., [17]), the frequency of use is estimated more accurately by counts from large longitudinal corpora.
- Special care is taken to partial out the effect of the sample length or the vocabulary size in parameters of the right truncated zeta distribution. We employed two different normalizations, one based upon the sample length [18], [19] and another based upon the observed vocabulary size. To our knowledge, the former is used for the first time in language acquisition research while the latter has never previously been considered in the language sciences.

However, our study restricts itself to humans in the hope of stimulating further cross-species research of the kind initiated in [14]. Here it will be shown that a constant value of 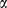 of 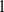 is unrealistic for speech in both children and adults. Furthermore, it will be shown that 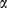 tends to decrease with age in many children while the trend in adults is weaker. Empirical evidence supporting a relationship between 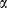 and MLU will also be provided. Despite its simplicity, MLU is a powerful estimator of syntactic complexity relying on the well-known fact that shorter sentences tend to be simpler ([9], pp. 82-83).

### The importance of text normalization

Our goal is to study the evolution of the exponent of Zipf's law during language ontogeny but we recognize that the exponent could be modulated or even determined by factors that are unrelated to the developmental stage. Therefore we address these issues upfront. For example, obvious variables such as the duration of the recording session or the amount of speech produced within a recording session of a given duration could be crucial artifacts in our analysis. However, concerning the latter, older children are expected to be able to produce more speech per unit of time than younger children. We illustrate a type of artifact that could occur due to undersampling: consider that the underlying distribution is such that 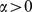. If the sample is short enough, repetitions of the same word may not occur (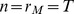) and the estimated 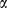 will be 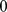 even though the true one is greater than zero. Indeed, the analysis of the text from the book €Alice in Wonderland€ suggests that 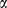 increases as a longer prefix of a novel is selected to estimate 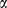 ([19], pp. 17-18), and even in large corpora the exponent of the law may depend on sample size [20], [21]. In our case, we are concerned about a possible dependency between 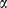 and 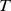, the total number of words of a sample on which the right-truncated zeta distribution is fitted. For this reason we employed a *length normalization*: for each individual and time point, a sample of 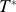 words is obtained (if 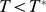 for that time point, then that time point is excluded in the subsequent analyses). We consider two different implementations of length normalization: *by prefix*, namely taking the 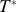 first word occurrences of the transcript or by *random sampling*, namely selecting 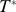 word occurrences uniformly at random from the whole sequence of the transcript. Normalization by prefix is equivalent to the normalization of [18], where participants are asked to speak for a total of 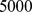 words (i.e. 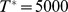). It could be argued that a normalization *by suffix*, namely taking the 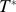 last word occurrences of the transcript should be considered as well but then the interpretation of results by suffix is harder because the properties of that suffix could have been determined by the part of the sequence that precedes the suffix but that is not analyzed. The goal of normalization by random sampling is to check if important information has been lost when considering the first words (and discarding the remainder), and also determining the extent to which the results depend on the use a prefix as well as establishing whether there could be other ways of obtaining similar results. For all these normalizations, two different cut-off values, 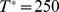 and 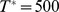 were selected (see Text S1 for a justification).

Another situation in which the exponent of Zipf's law could not be a direct assay of developmental stage is the following: the exponent is a mere by-product of the child's vocabulary size. Then, the exponent would not reflect any deep property of the lexicon or the overall organization of language. A variety of different methods have been developed to estimate actual vocabulary size: from parental language diaries through to vocabulary check-lists (see [17] and references therein). Unfortunately, such estimates are not easily available for the majority of children considered in our analysis (and the analysis becomes even more complex if one distinguishes between receptive and productive vocabulary [22]). However, we can use 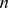, the number of different words that have appeared in a recording session as an estimate of the actual vocabulary size. Indeed, 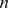 is the observed vocabulary size within a certain session. Thus, an *observed vocabulary size normalization* can be defined: for each individual and time point, a sample of 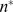 different words is obtained (if 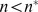 for that time point, then that time point is excluded in subsequent analyses). As is the case with length normalization, two different implementations of observed vocabulary size normalization can be used: *by prefix*, namely taking the smallest prefix of the transcript where 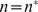 or by *random sampling*, in which word occurrences are selected uniformly at random from the whole sequence of the transcript till 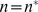. It is important be aware of an *a priori* independence between 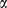 and 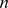. Since a maximum likelihood estimation procedure is used 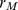 (the maximum rank) and 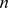 (the observed vocabulary size) coincide. The two parameters of the right-truncated zeta distribution that we fit, 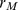 and 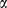, are independent parameters for the fitting procedure (only from a theoretical perspective as it is not entirely true that 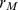 and 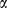 are independent *a priori*: 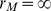 forces 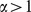, in practice only finite 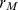 is supplied in a realistic fitting). *A priori*, Eq. 2 does not prohibit that the probability of a word (i.e. a rank) can become zero (decrementing 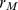) while 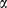 remains the same. Additionally, the probability of a word can change because another word is added (i.e., a word that had a probability of zero but now has a probability greater than one, thus incrementing 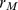) but 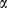 can remain the same (which happens when 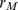 grows while 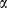 remains constant in a right-truncated zeta distribution). Nonetheless, it is still important to check that the amount of vocabulary observed in a session is not the factor that determines the evolution of the exponent of Zipf's law, and thus we examined two different cut-off values, 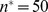 and 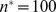 (see Text S1 for a justification).

Normalization by random sampling yields an unrealistic sequence of words (the words chosen are not necessarily consecutive in the original sequence of words) and thus the results of that analysis are presented in Text S1. However, it is important to evaluate whether the results of normalization by prefix are due to the realistic chain of words it forms.

We note various logical constraints in the application of these normalizations:

- A study of the correlation between mean length of utterance (MLU) and each of the two parameters of the right-truncated zeta distribution can only be carried out with normalization by prefix: normalization by random sampling is not concerned with the composition and length of utterances.
- In the context of normalization by prefix, the measurement of MLU is approximate. Consider the case of length normalization in which the last word of the 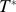 first words may not be the last word of a sentence. Therefore, we adopted the convention that the MLU of a certain prefix is the MLU over all the sentences that have at least one word in the prefix.
- Correlations between age or MLU and each of the two parameters of the right-truncated zeta distribution are correctly defined for length normalization but only correlations between age or MLU and 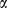 are valid for observed vocabulary size normalization. This is because observed length normalization imposes 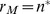 (i.e. 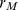 is constant), and therefore the correlation statistic is undefined.

## Results

The right-truncated zeta distribution was fitted to transcripts from longitudinal studies of child language from the CHILDES database [23]. The majority of corpora within this database are transcripts of conversational interactions among children and adults. Corpora that satisfied the following criteria were selected: they contained at least one target child for whom (1) there was a sufficiently large number of time points for a correlation analysis with age (see Methods) and (2) the crucial period between 1-3 years where multi-word utterances develop [9] was to a large extent covered. To keep the size of the dataset manageable, priority was given to corpora where it was indicated explicitly that the study was longitudinal or that the corpus was large (in terms of the number of time points) or dense (in proportion of time points within the time interval covered). Further details about the data analyzed are provided in the Methods section. Participants were classified into classes of role: target children (a target child is a child who was the focus of a study), fathers, mothers, investigators, other children, other adults and remainder (Table 1). Target children, fathers, mothers and investigators constitute what we the call major classes of roles. See the Methods section for further details.

**Table 1:** Mapping from CHILDES roles to our role classes.

Role	Role class
Adult	Other adults
Aunt	Other adults
Babysitter	Other adults
Brother	Other children
Camera operator	Other adults
Cousin	Other children
Child	Other children
Doctor	Other adults
Environment	Remainder
Family friend	Remainder
Father	Father
Girl	Other children
Grandfather	Other adults
Grandmother	Other adults
Investigator	Investigator
Mother	Mother
Non-human	Remainder
Observer	Other adults
Playmate	Other children
Sibling	Other children
Sister	Other children
Student	Remainder
Target child	Child
Teacher	Other adults
Therapist	Other adults
Toy	Remainder
Uncle	Other adults
Unidentified	Remainder
Visitor	Remainder

### The evolution of the parameters of Zipf's law

A global analysis of the correlation (Spearmans rank correlation [24]) between the parameters of the right-truncated zeta distribution and time was performed to study their evolution from two perspectives: the sign of the correlations (regardless of whether they are significant or not) and the sign and significance of the correlations. For a given language category, role class and parameter of the right-truncated zeta distribution, 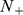 and 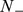 are defined as the number of individuals with a positive and negative correlation, respectively, while 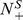 and 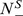 are defined as the number of individuals with a statistically significant positive and negative correlation respectively, and 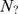 is the number of individuals with a correlation that is not significant.

**Figure 1:**
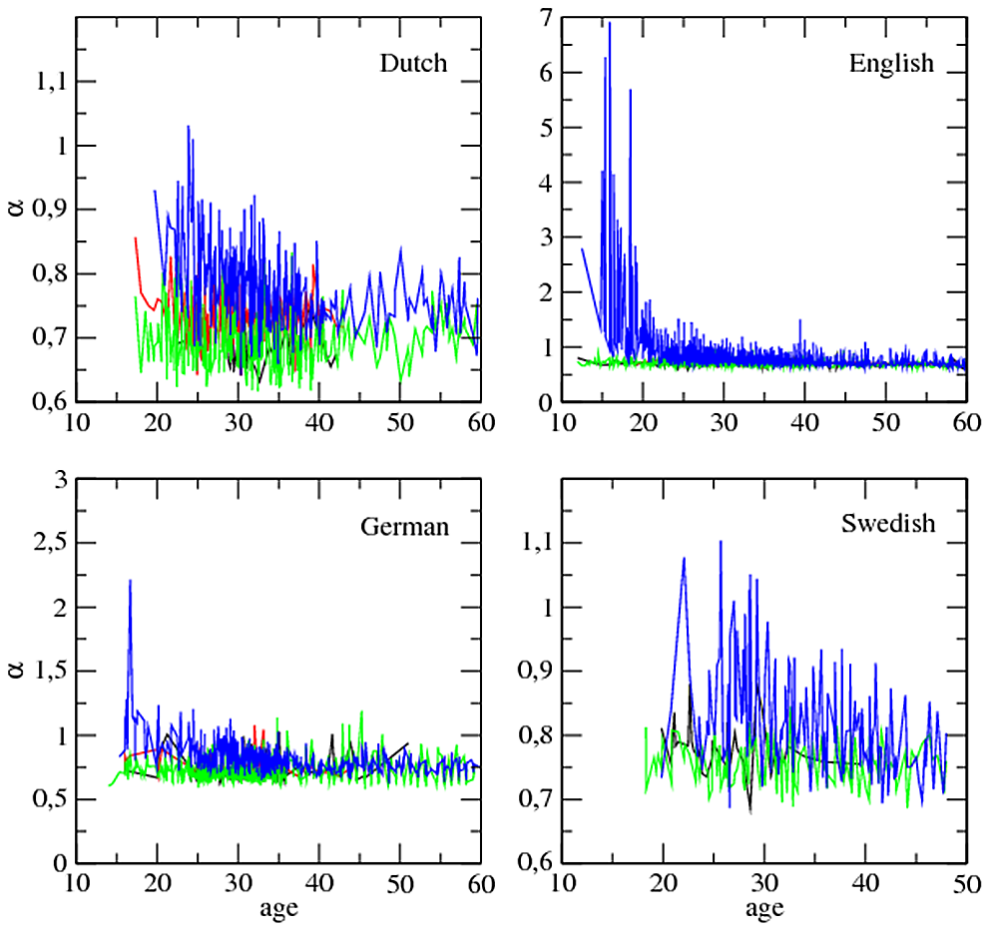
The evolution of the exponent 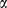 versus child age (in months): 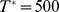. The major classes of roles, i.e. target children (blue), mothers (green), investigators (red) and fathers (black), are shown. Length normalization by prefix with 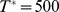 is used. Swedish lacks the class ‘investigator’.

**Figure 2:**
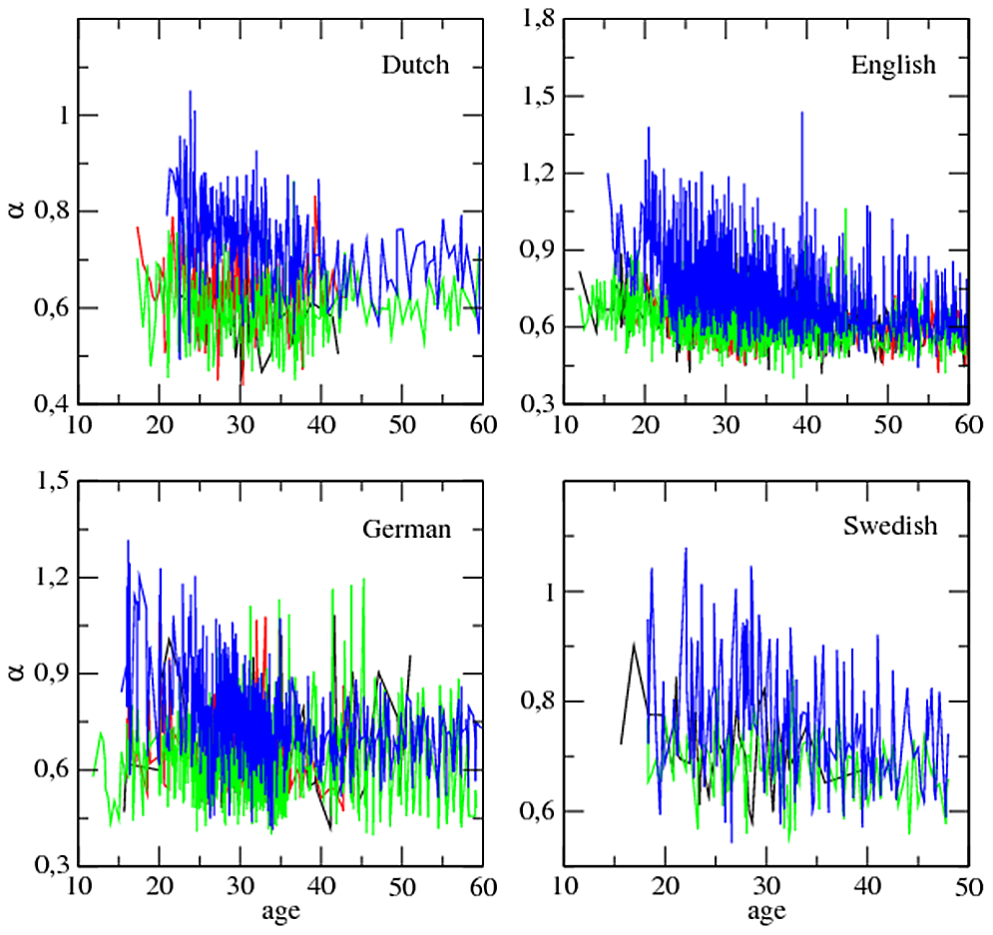
The evolution of the exponent 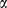 versus child age (in months): 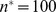. The major classes of roles, i.e. target children (blue), mothers (green), investigators (red) and fathers (black), are shown. Length normalization by prefix with 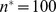 is used. Swedish lacks the class ‘investigator’.

**The evolution of** 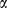. Figs. 1 and 2 show that 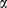 tends to decrease over time in the target children. A decline of 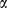 over time is also found in adults (e.g., mothers) but it is less pronounced or less clear than in the target children. Interestingly, 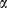 peaks between 15 and 20 months in English speaking children and less pronouncedly in German speaking children for length normalization (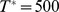 in Fig. 1; see also Text S1 for 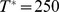). An analysis of the evolution of the exponent within each individual is necessary as the evolution in a mix of participants from a certain class of role may not be representative of the evolution in single participants from that class.

The analysis of the correlation between 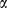 and time supports the idea that the behavior of infants and adults differs notably. The analysis of the sign of the correlation between 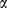 and age confirms the tendency of 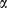 to decrease over time: 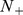 is never significantly high while 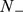 is significantly large in all target children with the only exception of Swedish speaking children, but we note that the number of Swedish target children is very small (Tables 2 and 3; similarly for lower cut-offs in Text S1 where the only exception are Dutch speaking children with 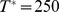). Additionally, 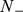 is also significantly large in investigators and parents in a certain language categories (English and ‘All’). If the significance of the correlation between 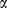 and age is taken into account, then it turns out that 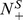 is very small (zero in the overwhelming majority of cases), and never significantly large (Tables 2 and 3; see also Text S1 for lower cut-offs). Interestingly, 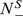 is significantly large for all target children (no exception), and the ratio 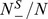 (where 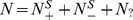) in target children is in stark contrast with that of other classes of roles where 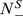 is significantly large. These results indicate that the decline of the exponent of 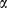 with time is stronger in children than in adults and suggests children are not simply mirroring the behavior of the adults with whom they are interacting. The range of variation 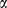 is consistent with this conclusion. If one focuses on the three major classes of roles: target children, investigators and parents, within a certain individual, (a) the maximum value of 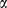 is maximum for children (b) the mean value of 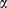 is also maximum for children (Tables 4 and 5; see also Text S1 for lower cut-offs).

**Table 2:** The dependency between 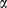 and age: length normalization by prefix with 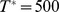.

Language	Role class	Sign of the dependency			Significance of the correlation			
		*N*	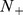	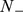	*N*	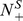	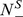	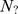
All	Target child	71	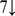	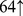	71	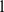	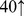	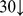
All	Father	14	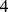	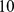	14	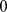	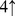	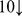
All	Investigator	17	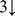	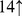	17	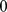	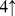	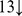
All	Mother	47	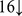	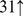	47	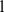	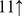	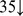
All	Other adults	8	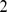	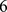	8	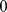	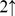	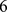
All	Other children	2	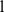	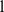	2	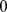	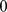	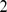
Dutch	Target child	12	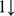	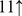	12	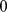	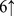	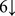
Dutch	Father	2	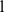	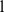	2	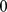	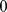	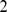
Dutch	Investigator	6	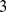	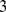	6	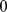	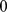	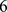
Dutch	Mother	7	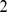	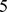	7	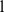	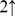	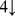
English	Target child	34	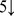	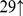	34	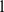	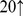	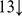
English	Father	7	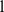	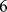	7	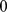	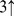	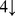
English	Investigator	8	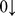	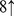	8	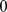	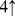	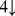
English	Mother	26	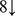	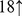	26	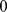	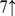	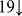
English	Other adults	2	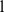	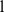	2	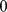	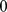	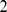
German	Target child	20	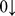	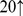	20	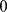	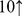	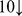
German	Father	3	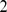	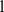	3	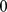	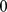	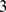
German	Investigator	3	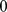	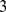	3	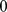	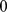	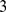
German	Mother	9	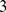	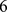	9	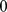	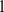	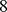
German	Other adults	3	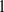	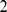	3	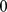	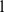	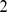
German	Other children	2	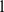	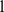	2	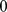	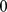	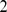
Swedish	Target child	5	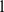	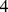	5	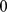	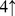	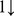
Swedish	Father	2	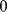	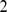	2	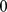	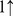	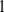
Swedish	Mother	5	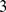	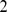	5	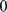	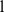	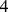
Swedish	Other adults	3	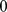	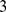	3	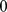	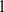	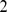

Analysis of the correlation between 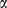 and age from two perspectives: the sign of the correlation and the significance of the correlations. Four language categories, i.e. All (all languages mixed), Dutch, English, German and Swedish, are considered. 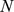 is the number of individuals analyzed for a given role class and language category that had at least 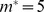 different points of time (the minimum number of points needed to show a significant correlation between a parameter and age through a two-sided correlation test at a significance level of 0.05, see the Methods section). This filter was applied for consistency between the analysis of the sign of the dependency and its significance. For each individual, the Spearman rank correlation [24] between age and a certain parameter of the right-truncated distribution was computed. In the analysis of the sign of the correlation, two counts are provided, namely 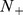 and 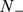, for each role class and language category. 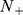 and 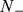 are, respectively, the number individuals with a positive and negative correlation (regardless of the sign of the correlation). In the analysis of the significance of the correlation, three counts are provided, namely 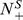, 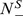 and 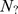, for each role class and language category. 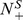 and 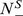 are the number individuals with a statistically significant positive and negative correlation, respectively. 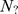 is the number of individuals with a correlation that is not significant. Significance was decided by a two-sided Spearman rank correlation test [24] at a significance level 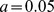. 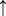 and 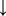 indicate counts that are, respectively, significantly high or significantly low according to a binomial test (see Methods).

**Table 3:** The dependency between 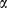 and age: length normalization by prefix with 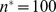.

Language	Role class	Sign of the dependency			Significance of the correlation			
		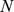	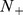	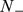	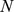	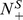	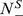	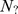
All	Target child	85	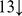	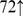	85	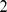	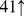	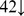
All	Father	19	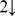	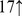	19	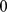	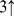	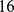
All	Investigator	25	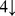	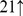	25	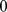	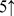	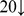
All	Mother	47	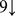	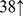	47	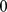	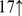	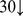
All	Other adults	15	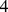	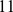	15	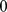	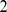	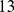
All	Other children	5	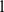	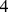	5	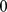	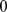	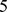
All	Remainder	1	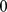	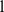	1	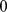	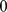	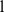
Dutch	Target child	14	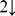	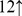	14	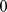	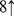	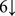
Dutch	Father	4	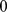	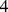	4	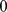	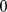	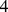
Dutch	Investigator	6	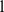	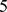	6	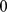	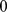	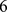
Dutch	Mother	7	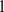	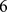	7	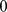	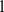	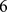
English	Target child	46	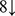	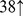	46	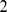	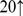	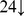
English	Father	10	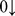	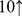	10	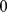	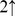	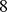
English	Investigator	15	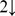	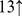	15	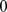	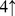	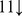
English	Mother	26	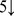	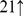	26	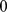	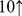	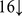
English	Other adults	8	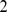	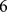	8	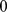	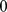	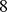
English	Other children	3	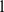	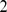	3	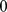	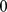	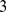
English	Remainder	1	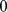	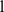	1	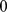	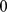	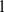
German	Target child	20	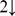	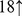	20	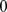	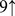	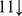
German	Father	3	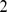	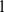	3	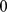	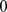	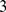
German	Investigator	4	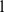	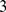	4	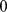	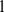	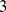
German	Mother	9	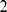	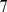	9	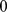	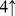	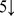
German	Other adults	4	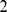	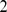	4	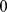	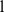	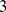
German	Other children	2	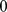	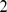	2	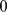	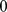	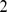
Swedish	Target child	5	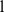	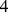	5	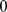	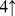	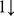
Swedish	Father	2	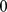	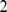	2	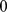	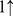	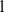
Swedish	Mother	5	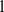	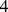	5	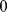	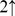	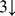
Swedish	Other adults	3	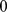	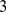	3	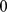	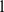	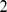

Methods (other than the normalization) and format are the same as in Table 2.

**Table 4:** Analysis of the variation the value of the exponent 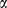: 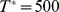.

Language	Role class	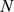	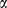			
			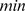	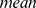	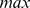	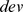
All	Target child	85	0.71 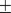 0.06	0.82 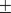 0.10	1.15 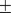 0.87	0.11 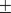 0.16
All	Father	21	0.67 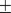 0.06	0.73 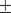 0.07	0.82 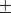 0.10	0.05 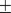 0.02
All	Investigator	21	0.68 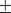 0.04	0.73 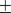 0.04	0.80 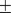 0.09	0.04 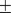 0.03
All	Mother	47	0.65 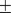 0.04	0.72 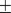 0.05	0.84 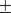 0.11	0.05 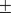 0.03
All	Other adults	17	0.71 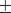 0.06	0.76 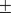 0.06	0.82 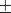 0.07	0.05 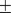 0.03
All	Other children	6	0.73 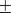 0.05	0.78 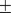 0.04	0.83 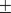 0.05	0.04 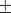 0.02
All	Remainder	1	0.67 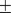 0.00	0.70 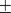 0.00	0.72 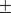 0.00	0.02 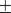 0.00
Dutch	Target child	14	0.72 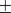 0.06	0.80 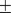 0.04	0.91 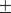 0.05	0.06 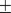 0.02
Dutch	Father	4	0.65 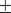 0.01	0.69 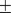 0.03	0.73 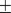 0.04	0.03 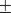 0.01
Dutch	Investigator	6	0.67 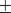 0.03	0.73 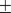 0.02	0.80 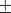 0.04	0.03 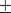 0.01
Dutch	Mother	7	0.63 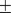 0.01	0.69 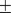 0.02	0.76 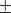 0.06	0.03 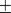 0.01
English	Target child	42	0.68 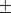 0.04	0.80 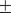 0.12	1.26 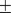 1.21	0.12 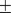 0.20
English	Father	11	0.65 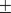 0.04	0.71 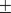 0.02	0.79 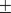 0.05	0.05 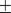 0.02
English	Investigator	10	0.66 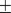 0.02	0.70 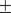 0.02	0.75 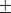 0.03	0.03 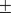 0.01
English	Mother	26	0.64 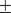 0.02	0.71 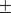 0.02	0.82 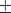 0.08	0.04 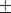 0.02
English	Other adults	8	0.69 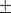 0.05	0.74 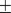 0.05	0.79 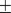 0.07	0.05 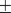 0.03
English	Other children	3	0.75 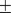 0.06	0.78 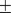 0.05	0.79 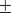 0.05	0.03 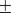 0.01
English	Remainder	1	0.67 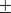 0.00	0.70 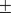 0.00	0.72 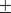 0.00	0.02 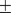 0.00
German	Target child	24	0.74 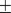 0.07	0.87 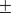 0.09	1.11 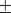 0.27	0.13 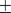 0.12
German	Father	3	0.71 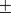 0.11	0.82 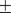 0.12	1.01 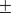 0.02	0.08 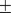 0.02
German	Investigator	5	0.72 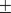 0.05	0.78 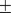 0.05	0.92 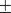 0.12	0.07 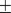 0.06
German	Mother	9	0.66 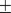 0.05	0.76 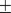 0.07	0.95 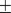 0.16	0.07 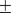 0.04
German	Other adults	5	0.70 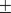 0.09	0.76 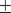 0.08	0.84 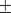 0.07	0.04 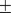 0.02
German	Other children	3	0.71 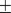 0.05	0.77 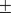 0.04	0.86 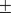 0.03	0.06 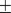 0.01
Swedish	Target child	5	0.71 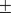 0.03	0.82 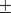 0.04	0.99 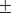 0.10	0.07 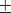 0.02
Swedish	Father	3	0.74 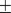 0.05	0.79 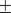 0.03	0.86 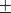 0.02	0.04 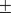 0.01
Swedish	Mother	5	0.71 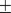 0.02	0.75 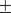 0.01	0.82 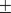 0.03	0.03 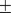 0.01
Swedish	Other adults	4	0.74 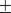 0.04	0.81 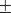 0.02	0.86 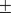 0.03	0.04 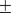 0.01

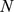
 is the number of individuals analyzed for a given role class and language category that have at least five time points (for consistency with the minimum number of points of the correlation analysis; see Methods). For each individual, four statistics concerning 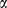 are computed: the minimum (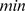), the mean (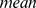), the maximum (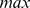) and the standard deviation (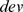) are calculated over all his/her transcripts. The mean plus/minus 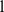 standard deviation of these four statistics is shown for each role class and language category (when 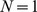, a standard deviation of 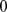 is assumed).

**Table 5:** Analysis of the variation the value of the exponent 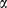: 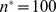.

Language	Role class	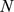	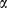			
			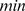	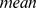	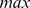	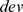
All	Target child	98	0.60 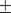 0.08	0.75 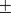 0.09	0.94 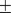 0.18	0.10 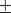 0.05
All	Father	22	0.53 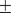 0.10	0.65 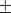 0.09	0.81 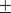 0.13	0.08 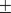 0.03
All	Investigator	39	0.54 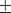 0.07	0.64 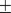 0.06	0.75 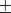 0.12	0.07 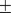 0.06
All	Mother	47	0.50 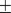 0.05	0.64 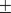 0.06	0.81 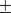 0.12	0.07 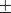 0.03
All	Other adults	26	0.57 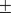 0.10	0.68 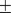 0.07	0.78 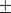 0.09	0.08 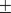 0.04
All	Other children	11	0.59 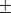 0.07	0.67 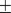 0.07	0.78 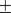 0.13	0.07 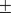 0.03
All	Remainder	2	0.67 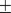 0.24	0.91 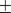 0.43	1.17 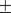 0.61	0.31 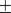 0.33
Dutch	Target child	14	0.63 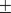 0.08	0.76 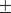 0.04	0.90 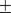 0.06	0.08 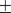 0.02
Dutch	Father	4	0.52 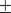 0.05	0.59 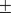 0.04	0.66 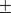 0.05	0.05 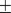 0.02
Dutch	Investigator	6	0.49 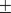 0.04	0.64 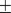 0.03	0.76 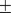 0.05	0.06 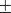 0.01
Dutch	Mother	7	0.48 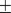 0.03	0.58 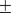 0.04	0.70 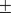 0.08	0.06 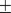 0.01
English	Target child	55	0.59 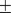 0.06	0.71 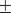 0.09	0.89 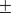 0.20	0.08 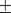 0.04
English	Father	11	0.49 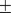 0.06	0.64 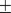 0.06	0.81 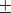 0.08	0.08 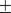 0.02
English	Investigator	24	0.55 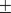 0.07	0.63 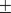 0.05	0.71 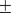 0.05	0.05 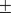 0.02
English	Mother	26	0.49 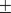 0.04	0.63 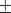 0.04	0.81 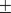 0.10	0.07 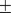 0.02
English	Other adults	17	0.57 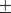 0.07	0.67 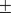 0.07	0.77 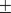 0.10	0.09 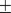 0.04
English	Other children	8	0.60 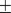 0.07	0.67 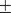 0.08	0.76 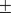 0.13	0.07 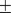 0.03
English	Remainder	2	0.67 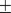 0.24	0.91 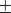 0.43	1.17 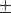 0.61	0.31 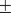 0.33
German	Target child	24	0.63 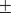 0.11	0.81 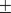 0.09	1.06 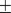 0.15	0.13 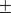 0.06
German	Father	4	0.55 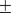 0.15	0.71 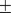 0.15	0.92 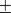 0.20	0.10 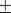 0.02
German	Investigator	9	0.56 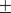 0.05	0.67 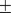 0.09	0.84 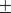 0.21	0.12 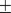 0.11
German	Mother	9	0.48 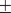 0.07	0.66 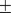 0.08	0.91 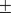 0.17	0.11 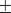 0.05
German	Other adults	5	0.50 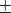 0.14	0.66 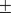 0.08	0.80 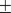 0.07	0.09 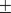 0.06
German	Other children	3	0.55 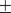 0.03	0.67 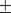 0.03	0.83 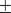 0.14	0.07 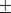 0.02
Swedish	Target child	5	0.59 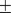 0.04	0.76 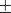 0.05	0.99 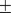 0.10	0.10 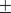 0.02
Swedish	Father	3	0.66 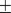 0.08	0.74 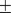 0.04	0.85 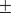 0.05	0.06 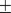 0.03
Swedish	Mother	5	0.58 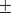 0.03	0.68 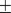 0.01	0.79 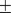 0.04	0.05 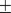 0.01
Swedish	Other adults	4	0.67 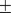 0.06	0.75 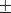 0.02	0.82 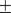 0.02	0.05 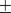 0.02

Observed vocabulary size normalization by prefix with 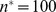 is used. The remainder of the methods and the format are the same as in Table 4.

**Figure 3:**
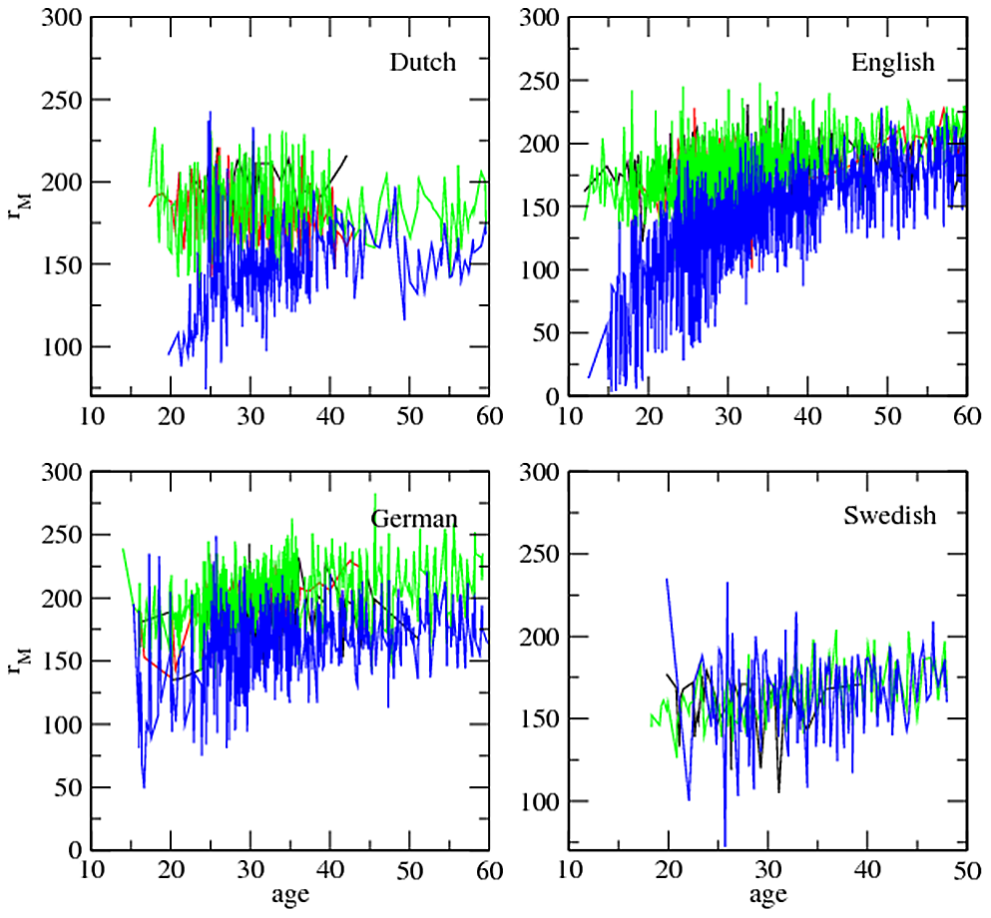
The evolution of the maximum rank 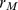
versus child age (in months):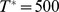. The major classes of roles, i.e. target children (blue), mothers (green), investigators (red) and fathers (black), are shown. Length normalization by prefix with 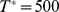 is used. Swedish lacks the class ‘investigator’.

**The evolution of** 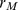. Excluding the peaks of 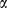 between 15 and 20 months mentioned above, the behavior of 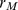 over time is the opposite to that of 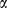. Fig. 3 shows that 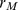 tends to increase over time in target children (see also Text S1 for a lower cut-off). An increase of 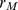 over time is also found in adults such as mothers but it is less pronounced or less clear than in target children.

The analysis of the correlation between 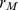 and time is not able to separate infants and adults as clearly as 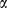 does. The analysis of the sign of the correlation between 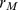 and age confirms the tendency of 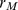 to increase over time: 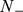 is never significantly high while 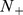 is significantly large in the majority of target children with the only exception of Swedish (recall that the number of target children is very small in that case), and also significantly large in investigators and parents depending on the language (Table 6; a lower cut-off in Text S1). The analysis of the significance of the correlation between 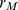 and age reveals that 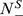 is very small (zero in the majority of cases), and never significantly large (Table 6; see also Text S1 for a lower cut-off). Interestingly, 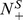 is significantly large for all target children (Swedish being the only exception). With regards to 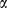 versus time, the ratio 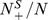 is more balanced between target children and the adults where 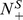 is significantly large in some case (e.g., mothers). These results indicate that the increase of 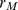 with time does not distinguish children from adults as clearly as 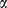 in terms of the relative proportion of individuals who show a negative correlation but recall that the increase of 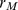 is more pronounced in children (Fig. 3 and Text S1.)

**Table 6:** The dependency between 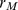 and age: length normalization by prefix with 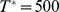.

Language	Role class	Sign of the dependency			Significance of the correlation			
		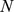	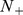	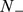	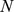	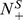	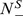	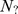
All	Target child	71	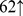	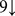	71	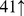	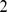	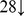
All	Father	14	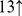	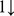	14	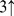	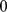	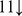
All	Investigator	17	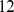	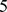	17	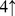	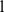	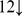
All	Mother	47	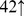	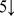	47	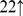	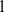	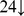
All	Other adults	8	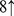	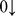	8	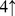	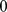	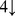
All	Other children	2	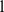	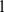	2	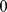	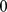	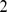
Dutch	Target child	12	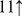	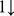	12	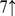	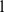	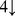
Dutch	Father	2	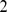	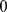	2	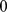	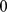	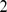
Dutch	Investigator	6	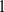	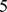	6	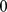	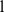	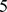
Dutch	Mother	7	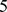	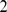	7	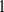	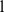	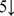
English	Target child	34	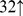	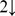	34	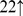	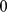	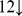
English	Father	7	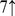	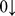	7	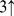	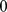	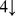
English	Investigator	8	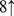	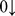	8	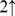	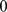	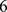
English	Mother	26	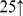	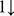	26	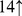	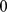	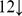
English	Other adults	2	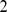	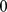	2	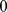	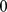	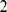
German	Target child	20	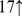	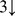	20	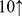	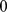	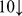
German	Father	3	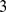	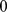	3	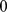	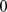	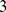
German	Investigator	3	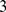	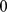	3	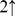	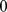	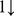
German	Mother	9	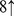	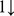	9	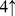	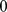	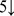
German	Other adults	3	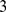	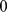	3	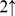	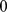	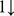
German	Other children	2	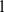	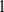	2	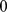	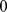	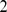
Swedish	Target child	5	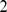	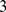	5	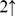	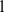	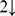
Swedish	Father	2	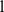	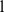	2	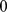	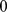	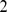
Swedish	Mother	5	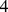	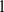	5	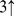	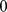	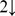
Swedish	Other adults	3	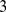	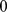	3	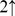	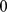	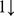

Methods (other than the target parameter) and format are the same as in Table 2.

### The relationship between the exponent of Zipf's law and the mean length of utterances

**Figure 4:**
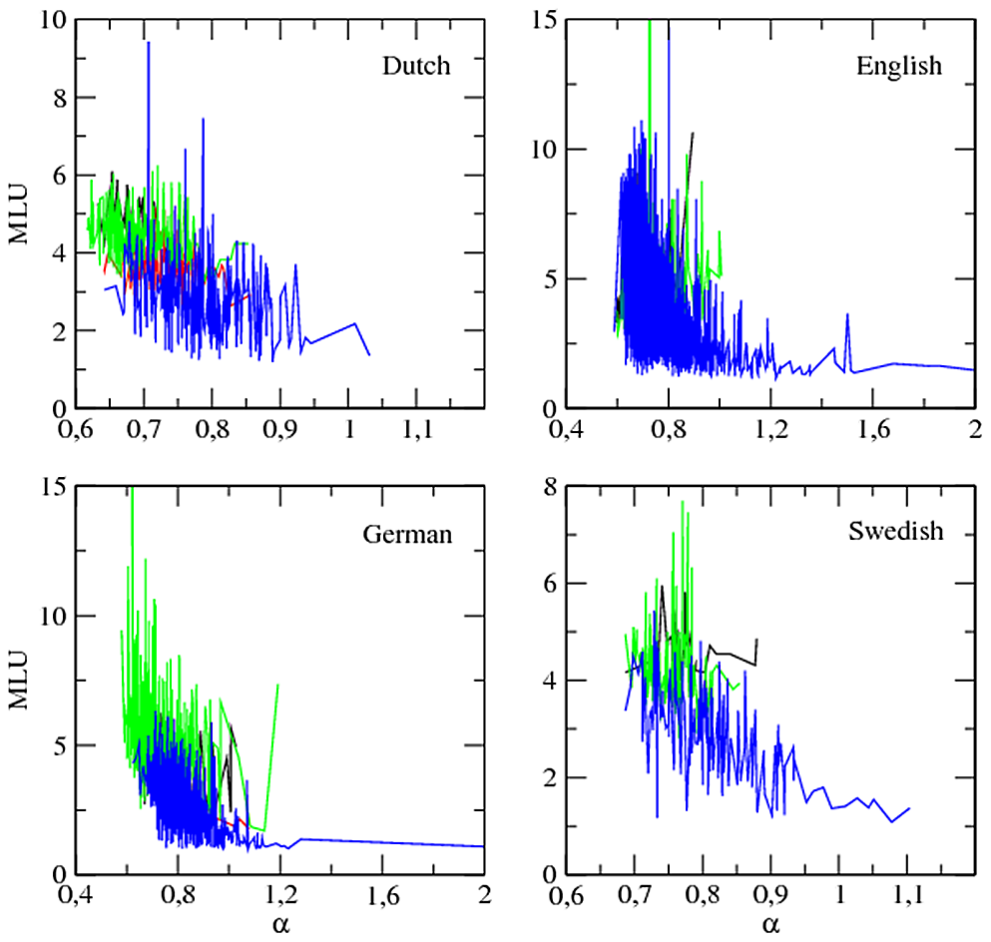
The MLU (in words) versus 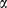 : 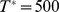. The major classes of roles, i.e. target children (blue), mothers (green), investigators (red) and fathers (black), are shown. Length normalization by prefix with 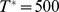 is used. Swedish lacks the class ‘investigator’. In order to facilitate the visual inspection of the series, the few points with MLU above 15 or 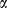 above 2 are not shown (this concerns English and German).

**Figure 5:**
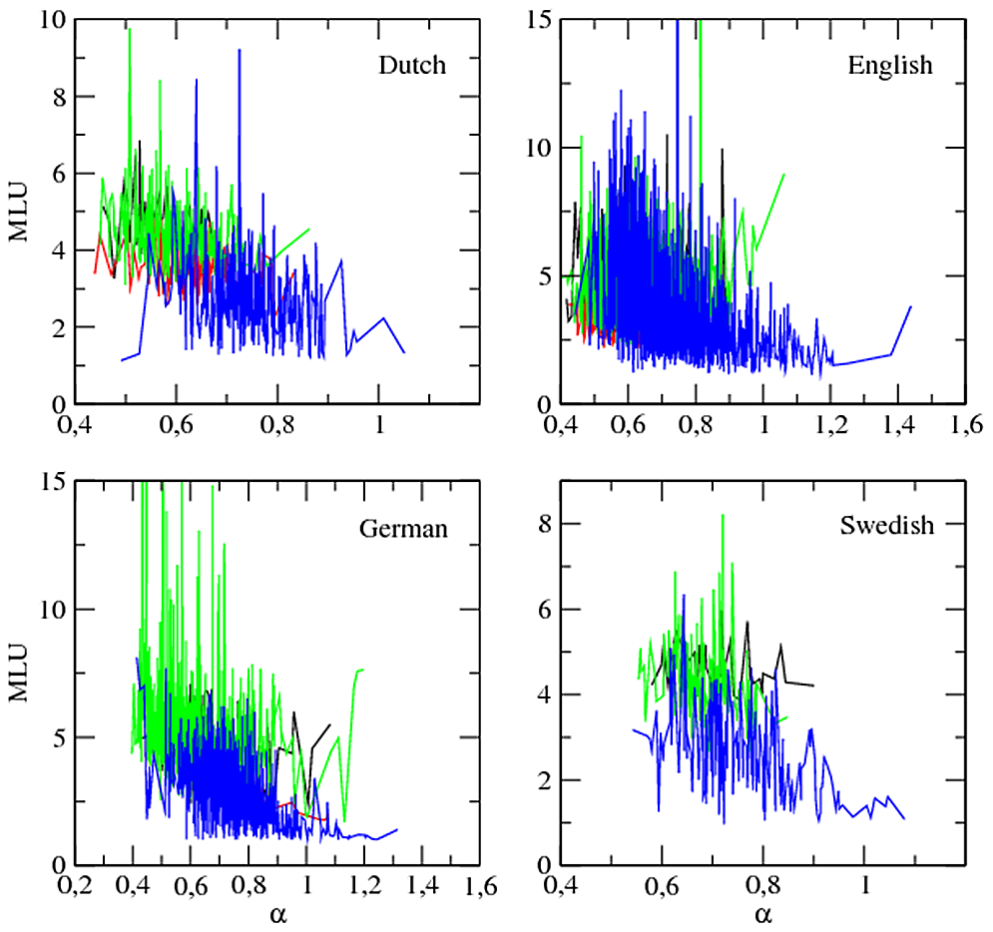
MLU (in words) versus the exponent 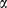: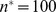. The major classes of roles, i.e. target children (blue), mothers (green), investigators (red) and fathers (black), are shown. Length normalization by prefix with 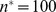 is used. Swedish lacks the class ‘investigator’. In order to facilitate the visual inspection of the series, the few points with MLU above 15 are not shown (this concerns English and German).

Figs. 4 and 5 show that MLU tends to increase as 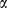 decreases at least for target children (see also Text S1 for plots with lower cut-offs). However, an analysis of each individual within each class, as we did for the parameters of Zipf's law and time, is necessary. Here, the meaning of 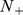, 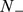, 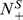, 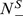 and 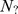 is modified slightly. Instead of referring to correlations with age, they refer to correlations with mean length of utterance (MLU) in words. The analysis of the sign of the correlation between MLU and 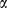 (regardless of whether it is significant or not) reveals that 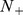 is never significantly high for all classes of roles but that 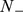 is significantly high for target children in the majority of cases (it fails when 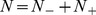 is small, namely in Swedish) while it is occasionally significant for investigators and other adults (Table 7 for length normalization and Table 8 for observed vocabulary size normalization; see also Text S1). As in the case of the evolution of 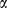 with time, these results suggest that children are not mirroring the behavior of the adults with whom they are interacting.

**Table 7:** The dependency between 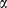 and MLU: length normalization by prefix with 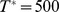.

Language	Role class	Sign of the dependency			Significance of the correlation			
		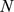	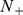	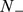	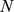	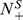	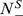	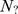
All	Target child	71	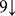	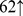	71	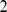	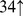	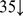
All	Father	14	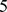	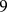	14	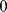	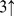	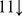
All	Investigator	17	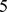	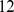	17	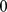	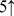	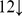
All	Mother	47	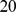	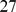	47	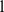	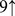	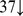
All	Other adults	8	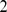	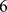	8	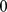	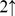	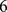
All	Other children	2	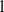	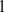	2	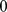	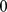	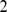
Dutch	Target child	12	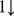	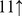	12	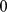	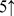	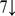
Dutch	Father	2	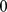	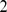	2	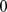	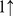	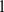
Dutch	Investigator	6	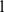	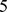	6	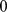	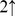	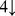
Dutch	Mother	7	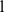	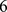	7	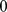	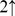	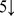
English	Target child	34	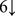	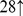	34	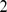	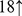	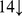
English	Father	7	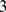	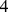	7	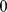	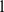	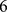
English	Investigator	8	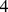	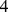	8	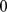	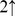	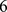
English	Mother	26	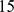	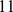	26	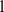	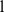	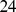
English	Other adults	2	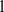	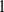	2	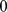	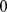	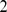
German	Target child	20	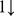	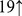	20	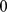	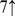	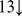
German	Father	3	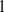	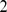	3	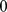	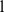	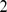
German	Investigator	3	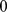	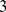	3	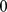	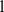	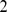
German	Mother	9	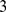	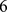	9	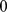	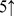	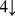
German	Other adults	3	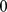	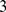	3	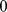	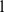	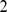
German	Other children	2	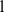	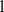	2	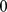	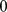	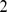
Swedish	Target child	5	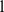	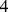	5	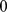	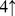	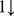
Swedish	Father	2	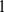	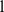	2	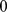	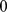	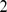
Swedish	Mother	5	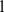	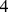	5	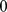	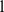	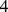
Swedish	Other adults	3	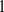	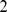	3	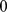	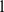	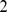

Methods (other than the target variables) and format are the same as in Table 2.

**Table 8:** The dependency between 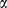 and MLU: length normalization by prefix with 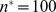.

Language	Role class	Sign of the dependency			Significance of the correlation			
		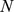	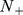	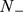	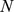	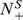	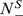	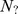
All	Target child	85	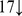	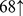	85	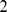	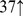	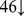
All	Father	19	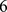	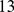	19	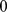	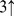	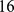
All	Investigator	25	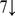	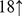	25	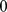	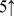	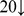
All	Mother	47	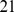	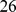	47	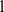	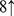	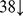
All	Other adults	15	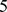	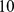	15	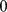	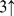	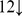
All	Other children	5	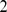	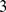	5	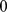	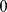	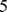
All	Remainder	1	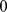	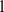	1	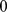	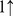	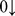
Dutch	Target child	14	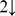	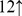	14	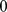	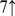	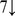
Dutch	Father	4	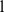	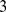	4	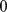	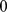	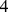
Dutch	Investigator	6	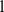	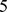	6	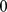	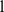	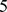
Dutch	Mother	7	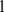	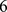	7	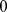	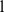	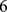
English	Target child	46	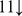	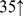	46	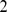	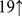	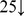
English	Father	10	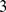	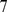	10	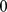	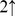	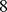
English	Investigator	15	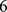	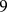	15	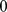	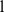	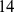
English	Mother	26	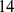	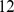	26	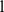	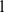	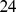
English	Other adults	8	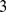	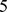	8	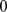	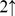	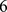
English	Other children	3	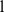	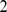	3	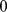	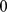	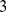
English	Remainder	1	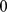	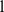	1	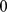	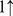	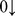
German	Target child	20	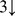	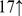	20	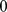	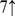	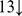
German	Father	3	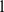	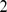	3	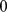	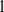	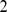
German	Investigator	4	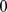	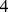	4	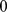	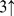	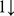
German	Mother	9	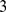	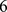	9	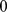	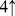	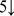
German	Other adults	4	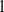	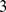	4	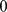	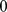	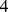
German	Other children	2	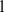	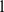	2	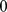	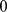	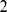
Swedish	Target child	5	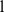	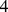	5	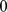	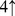	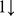
Swedish	Father	2	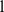	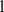	2	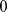	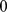	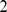
Swedish	Mother	5	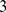	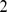	5	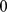	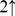	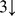
Swedish	Other adults	3	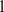	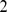	3	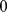	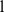	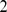

Methods (other than the normalization and the target variables) and format are the same as in Table 2.

The analysis of the significant correlations between MLU and 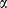 reveals that 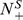 is never significant for all classes of roles (Table 7 for length normalization and Table 8 for observed vocabulary size normalization) with the only exception of a few English mothers (see Text S1). 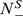 is significantly high in all target children while less frequently in other classes of roles. Interestingly, 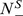 cannot be explained, in general, by a transfer from adult speech to children. For instance, when all languages are mixed the sum of 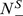 of parents, investigators and other adults yields 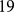 (Table 7 and Table 8) while target children go further: 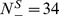 with 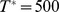 (Table 7) and 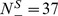 with 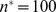 (Table 8). These findings suggest again that the negative correlation between MLU and 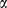 in children is not a simple mirror of adult behavior.

In sum, the number of positive correlations between MLU and 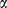 (significant or not) is never significantly high. There is a clear bias for negative correlations between MLU and 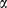, specially in target children.

## Discussion

The idea that Zipf's law for word frequencies is a power law with a constant exponent of 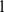, independently of linguistic complexity, needs to be revised [3], [8]. Our conclusion is derived from several sources: the dependency of the exponent with time, the value of the exponent, and the relationship between the exponent and linguistic complexity.

### The evolution of the exponent

Figs. 1 and 2 (also Text S1) indicate that children evolve from a high value of 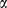 to the value of 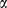 of adults at least from about 20 months onwards (recall that some normalizations suggest a peak of 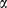 between 15 and 20 months in children who speak English or German). Importantly, the evidence concerning the tendency of the exponent of Zipf's law to evolve in children (Tables 2 and 3; see also Text S1) indicates that Zipf's law is not a static property of language as many models of the evolution of language assume [3], [6]–[8].

### The value of the exponent

The dependency of 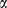 with time not only contradicts the assumption of a constant exponent but also the value of the exponent itself. Both in adults and children the exponents are on average below 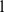 (Tables 4 and 5; see also Text S1) which is the typical value assumed, or used, to define the law [3], [4]. For target children, the mean exponent is 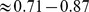 (Table 4 and 5; see also Text S1). Interestingly, the mean exponents of the main adult roles are bounded above by the exponents of target children. The standard values assumed for the exponent of Zipf's law, at least in adult speech, needs to be reconsidered. A complementary analysis of the variation of 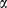 is reported in Text S1. Further support for 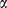 as a free parameter of Zipf's law comes from a comparison of the fit of the truncated zeta distribution, which has two parameters, 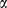 and 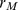, and a simplified version with 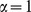 and only one parameter, i.e. 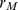 (Text S1). The comparison suggests that the version with two parameters is a superior model of word frequencies in the overwhelming majority of cases even when a penalty for the number of free parameters (a reward for parsimony) is applied to evaluate the quality of the fit.

The standard assumption of a value of 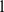 for the exponent of Zipf€s law may have endured because the vast majority of research on Zipf's law exploits large literary texts [1], [25] (simply due to their availability), as well as the manner in which Zipf's law traditionally has been studied [1], [25]. Concerning the latter, large texts are needed to uncover a straight-line in double logarithmic scale over many decades and then be able to (a) conclude that Zipf's law holds approximately according to a visual test or (b) estimate the exponent. In contrast, the CHILDES transcripts provide samples that are too small for the traditional visual approach, namely plotting the empirical rank distribution in double logarithmic scale and concluding that the law holds if the distribution appears as a long straight line. Also, there is a growing consensus on the superiority of the estimation of the exponents of power laws by maximum likelihood over traditional methods even in small samples [16], [26] such as the transcripts from individual recording sessions in the CHILDES database. The combination of powerful methods such as maximum likelihood [15] and electronic databases of speech such as CHILDES [23] may challenge traditional notions of Zipf's law and its parameters. However, the effect of size and modality (oral versus written) on Zipf's law needs further investigation. Another important issue for future research is the possibility that the exponents of adults are not a genuine manifestation of adult speech but a consequence of a series of adaptations to children at many levels, namely phonology, vocabulary, morphology and syntax, that are known as child-directed speech [9]. Furthermore our findings suggest that another aspect should be considered in child-directed speech: the patterning of word frequencies. A tendency of 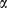 to decrease with time has been found in children but to a substantially lesser degree in adults. This tendency in adults could be a manifestation of the adaptation of some adults to child behavior at the level of word frequencies. Clearly further research is necessary.

### The relationship between the exponent and linguistic complexity

Crucially, our findings provide support for the hypothesis that the exponent of Zipfs law might be intimately related with the complexity of the actual communication system [10], [11]. According to the €language for free hypothesis [10], , (1) a rudimentary form of language (including a rudimentary form of syntax and symbolic reference) as well as various statistical patterns of language (such as the degree distribution of word-word interactions) could be a by-product of Zipf's law with a particular exponent and (2) Zipf's law could in turn be a by-product of general communication principles [10], [11]. Our finding of the tendency of 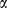 to decrease as MLU (a simple indicator of syntactic complexity) increases provides empirical support for the abstract information and network theoretic arguments used to sustain the dependency between 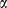 and language complexity of this hypothesis [10], [11]. Models of the evolution of language in children assuming a constant exponent [8] are clearly in need of revision (see Tables 4 and 5 and Figs. 1 and 2; also Text S1) that we take to suggest that the assumption of a constant exponent is more appropriate for the speech of adults than for the speech of infants.

It is tempting to believe that the tendency of the exponent of Zipf's law to decrease as a simple indicator of syntactic complexity (MLU) increases occurs simply because of two facts: the established tendency of MLU to increase as children grow older [9], [22], [28] and the tendency of 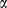 to decrease as children grow older (as reported in the present article). However, a correlation is not transitive in the sense that a correlation between 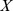 and 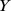 and a correlation between 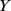 and 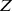 does not imply a correlation between 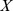 and 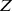 [29]. Nonetheless, the depth of the inverse relationship between MLU and the exponent of Zipf's law, such as the weight of the contribution of the exponent, age and other factors in determining MLU, should be investigated.

### Towards the future

We have considered a very simple case of the evolution of the exponent of Zipf's law with age: a monotonic increase or decrease, which is the sort of dependency that the non-parametric correlation test we have employed is able to detect. Future work needs to address other forms of dependency between the exponent and time, such as a bell-shape (a growth of 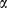 with time followed by a decrease) that has been suggested by cross-species studies in the development of repertoires by means of broad age groups[14], or oscillatory convergence. Visual support for the hypothesis of a bell-shape comes from normalization by prefix with 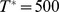 and 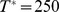 in English (Fig. 1 and Text S1, respectively), with 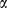 peaking between 15 and 20 months of age. However, this pronounced peak weakens when considering the normalization by prefix with 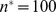 and 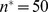 (Fig. 2 and Text S1, respectively). Visual support for a bell-shape in other languages is less clear but this could be simply because in our analysis English is the largest and most extensive dataset (see Methods and Text S1). Thus we acknowledge that our work constitutes only the preliminary step towards a full understanding the evolution of 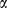. The hypothesis of a bell-shape needs further examination.

Our selection of a right-truncated zeta distribution was motivated by the choice that models of language evolution had previously adopted [3], [8]. Other probability distributions are known to be capable of giving a better fit to literary writings and other ‘texts’ than a right-truncated zeta distribution (e.g. [12], [30]). Models of the evolution of language that are based on a power law with an exponent 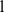 add yet further challenge for future research, namely exploring the effect of more realistic exponents (e.g. time-dependent exponents) or alternative distributions.

## Materials and Methods

### The dataset

The longitudinal studies of child language development from the CHILDES database [23] that were employed are:

- *Dutch (14 target children)*: Groningen Corpus [31] (6 target children), Schaerlekens Corpus [32] (6 target children) and van Kampen Corpus [33] (2 target children). As for the Groningen Corpus, ‘Iris’ was removed because she subsequently displayed delay in language development due to hearing problems. ‘Iri’ (ending with no ‘s’) was also excluded (this person was very likely a misspelling of ‘Iris’ because he/she was in the same subdirectory of ‘Iris’ and was the only target child in the only file where it appeared).
- *English (60 target children)*. In the case of British English, the Lara Corpus [34] (1 target child), the Manchester Corpus [35] (12 target children), and the Wells Corpus [36] (32 target children) were used. For American English, the following corpora were used: Bloom 1970 Corpus [37]–[39] (2 target children; Gia was excluded because age information is not reported for her), Brown Corpus [40] (3 target children), Kuczaj Corpus [41] (1 target child), MacWhinney Corpus [42] (2 target children), Providence Corpus [43] (5 target children; Ethan was excluded because he was diagnosed with Asperger's Syndrome at the age of 5 [42]), Sachs Corpus [44] (1 target child) and Suppes Corpus [45] (1 target child).
- *German (26 target children)*: Caroline Corpus [46] (1 target child), Leo Corpus [47] (1 target child), Rigol Corpus [46] (3 target children) and Szagun Corpus [48] (21 target children). For the Szagun Corpus, only the normally hearing children, i.e. Ann, Eme, Fal, Lis, Rah and Soem, were used (the children with cochlear implants were excluded).
- *Swedish (5 target children)*: Goteborg Corpus [49], [50] (a file contains one more target child, Eva, who does not speak at all).

All the corpora of the CHILDES database are freely available at *http://childes.psy.cmu.edu/data/*(accessed 17 December 2012). Some corpora that we employed contain target children with names that do not match any of the target children names provided in the CHILDES database documentation [51]. All these anomalous cases appear in only one file and thus there is only one time point for them. All these children were removed. Time points for which age was not provided or was clearly incorrect were removed prior to analysis. Therefore the whole Thomas corpus of British English [52] could not be included in our study.

An upper limit of 5 years was chosen to avoid the possibility that significant correlations with age do not surface because the child's vocabulary usage has converged to some stationary state. Additionally, the exclusion of materials from five years onwards is important for the Rigol Corpus [46] which contains transcriptions of elicitation tasks that deviate from a typical spontaneous linguistic interaction of the CHILDES database from five years onwards. A summary of the age ranges of the target children included in our analysis is provided in Text S1.

In order to summarize results in a homogeneous and compact fashion the roles adopted in the CHILDES database were grouped into classes. Table 1 shows the correspondence between CHILDES roles and our role classes. Table 9 shows that the roles target child, father, mother and investigator cover the overwhelming majority of words produced in each language category. For this reason, the remaining roles were classified into three broad role classes: ‘other children’, ‘other adults’ and ‘remainder’. A principle of design of this classification was to facilitate the study of the evolution of Zipf's law homogenously across languages taking into account the different ways in which the speech of children and adults can manifest [53]. The classes ‘father’ and ‘mother’ could be replaced by a class parents since in general fathers contributed less than mothers and proportionally little with regard to all classes. Curiously, fathers and mother contributed an approximately similar amount in Swedish, and an homogeneous categorization across languages was a design concern (Table 9). Furthermore, language acquisition research suggests that fathers produce a kind of child-directed speech that is less finely tuned to the child's developmental level than do mothers (see [53] and references therein) and we aim to investigate if the evolution of Zipf's law in children could be a simple mirror of adult speech, or child-directed speech, a specific form of speech directed to children by adults [53]. The class ‘target child’ and ‘other children’ could also be mixed but that could imply mixing children at radically different developmental stages and even siblings of target children could be showing a muted form of child-directed speech [53]. This was a further reason not to remove the class ‘other children’ from the analysis (notice that CHILDES, in general, does not report the age of children who do not take the role ‘target child’). The fact that individuals falling in the category ‘other adults’ may be showing a very smoothed version of child-directed speech with regards to parents (or even no child-directed speech at all) motivated us to keep the class for reporting results although it has a low weight in the dataset Table 9. The class ‘remainder’ was added for completeness.

**Table 9:** Proportion of words produced within each role class as a function of language.

Language	Role class	Proportion of words.
All	Target child	47.66
All	Father	6.23
All	Investigator	8.87
All	Mother	30.01
All	Other adults	5.61
All	Other children	1.36
All	Remainder	0.25
Dutch	Target child	38.58
Dutch	Father	5.82
Dutch	Investigator	30.18
Dutch	Mother	25.39
Dutch	Other children	0.02
English	Target child	42.24
English	Father	6.96
English	Investigator	7.46
English	Mother	37.59
English	Other adults	4.06
English	Other children	1.29
English	Remainder	0.40
German	Target child	69.03
German	Father	2.17
German	Investigator	2.07
German	Mother	16.08
German	Other adults	7.77
German	Other children	2.78
German	Remainder	0.10
Swedish	Target child	41.97
Swedish	Father	14.44
Swedish	Mother	19.83
Swedish	Other adults	23.76

Role classes without words are omitted.

Before applying the conversion to role class, the following preprocessing was performed:

- Concerning the Lara Corpus, the only child appearing with the role ‘Child’ was assigned the new role ‘Target child’.
- All individuals from the same corpus with the same role who did not have a name were treated as the same individual.
- The MacWhinney corpus is split into parts. Such subdivision was not taken into account. All the transcripts were used regardless of the subcorpus they belonged to.

All tokens were lower-cased. Raw word forms were used (lemmatization was not applied).

### The fit of a right-truncated zeta distribution

The right-truncated zeta distribution was fitted by maximum likelihood [15], namely the parameters of the function were obtained by maximizing a log-likelihood function that is presented next. We define 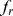 as the frequency of rank 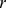 in a text and 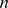 as the number of different words of that text. 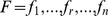 defines the rank histogram of a text. The likelihood of 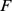 can defined as [15]

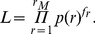
(4)


Taking logs on both sides of the previous equation we obtain the log-likelihood, namely
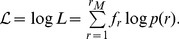
(5)


Replacing the definition of the right-truncated zeta distribution in Eq. 2 into Eq. 5, yields
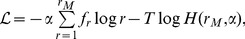
(6) where
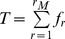
(7) is the text length in words.


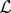
 was maximized using a quasi-Newton method that allows one to define upper and lower bounds to parameters [54]. 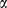 was restricted to the interval 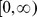, which follows by the definition of rank (the probability of a rank cannot increase as rank increases). 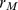 was restricted to the interval 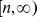 as 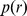 is non-zero if and only if 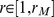 and values of 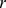 that have occurred in the text at least once cannot have a zero probability of occurring. The initial values of 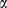 and 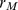 were 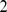 and 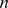 respectively.

### Filtering of data

For a given individual, samples containing only one different word (no matter how many times this word was produced) were excluded from our analyses. When a sample has only one different word then the exponent 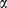 cannot be estimated properly. In this case, 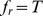 if 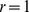 and 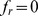 otherwise, and thus Eq. 6 becomes
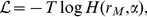
(8) which is maximized when 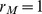 given 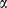 but 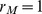 yields 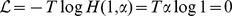, which means that 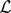 achieves its theoretical maximum regardless of the value of 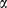.

Depending on the kind of analysis further constraints were imposed. In Tables 2, 3, 6, 7 and 8 (and similar tables in Text S1), all participants with a number of time points smaller than 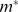 were excluded from the analyses. 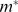, the minimum number of points that are needed by a two-sided correlation test between two vectors 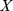 and 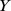, is the smallest value of 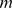 satisfying the condition [55]

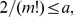
(9) where 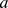 is the significance level and the factor 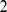 is the number of permutations of 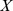 that yield a correlation as large (in absolute value), as that of 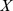 and 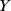 in the original order. The factor 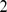 comes from the fact that 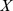 and the reverse of 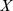 give a correlation whose absolute value is as large as that of the original 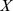). With 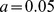 then 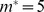.

### Binomial tests


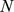
 is defined as the number of individuals with at least 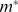 points of time, 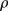 as the Spearman rank correlation and 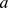 as the significance level of that test. Under the null hypothesis,

- The probability that 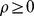 is 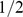, which implies that 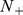 and 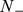 follow a binomial distribution with parameters 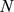 and 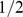.
- The 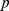-values of a continuous statistic are known to be uniformly distributed [56]. In our case, 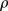 is approximately continuous and the quality of the approximation increases as 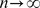. This implies that 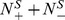 follows a approximately a binomial distribution with parameters 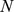 and 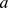 whereas 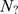 follows approximately a binomial distribution with parameters 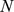 and 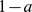. Recalling that the probability that 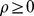 is 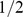 under the null hypothesis, it is obtained that 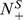 and 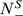 follow approximately a binomial distribution with parameters 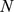 and 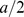. Notice that individuals who cannot yield a 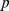-value equal smaller than 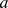 have been excluded in the analysis of the significance of 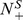, 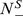 and 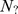.

In sum, whether 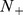, 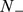, 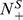, 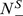 and 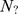 are significantly high or low can be assessed by means of binomial test with the parameters of the distribution indicated above [24]. Such binomial tests were used for computing the 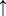 and 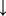 arrows in Tables 2, 3, 6, 7 and 8 (and also similar tables in Test S1).

## Supporting Information

Text S1
**It shows the age ranges of the target children considered for our analysis, explains the rationale behind the choice of the different cut-offs, shows results not included in the main article (based upon lower cut-offs for normalization by prefix and also the normalization by random sampling, which is not used for the main article), compares the fit of a fixed** 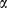 **(** 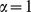 **) versus a free** 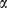 **and summarizes the range of variation of the exponent** 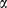 **.**
(PDF)
