# Supplementary material for: The Evolution of the Exponent of Zipf's Law in Language Ontogeny
Source: PLoS One. 2013 Mar 13;8(3):e53227. doi: 10.1371/journal.pone.0053227 (PMC3596411; doi:10.1371/journal.pone.0053227)
Supplement: Text S1 — It shows the age ranges of the target children considered for our analysis, explains the rationale behind the choice of the different cut-offs, shows results not included in the main article (based upon lower cut-offs for normalization by prefix and also the normalization by random sampling, which is not used for the main article), compares the fit of a fixed ( ) versus a free and summarizes the range of variation of the exponent . (PDF) [file pone.0053227.s001.pdf]

Supplementary online information for ’The evolution of the exponent of Zipf’s law in language ontogeny’

Jaume Baixeries[1,2], **Brita Elvevåg**[3,4], **Ramon Ferrer-i-Cancho**[2]‡

[1] LARCA Research Group
Departament de Llenguatges i Sistemes Informàtics,
Universitat Politècnica de Catalunya,
Barcelona, Catalonia (Spain)

[2] Complexity & Quantitative Linguistics Lab
Departament de Llenguatges i Sistemes Informàtics,
TALP Research Center, Universitat Politècnica de Catalunya,
Barcelona, Catalonia (Spain)

[3] Psychiatry Research Group,
Department of Clinical Medicine,
University of Tromsø,
Tromsø, Norway

[4] Norwegian Centre for Integrated Care and Telemedicine (NST),
University Hospital of North Norway,
Tromsø, Norway.

‡ Corresponding author: rferrericancho@lsi.upc.edu.

Contents

1 Age ranges of target children **3**

2 The cut-offs for normalization **6**

3 Normalizations excluded from the main article **8**
3.1 Normalization by prefix: additional tables with lower cut-offs 8
3.1.1 Dependencies of parameters with age 8
3.1.2 Dependencies between α and MLU 15
3.2 Normalization by random sampling. 19
3.2.1 Dependency of parameters with age 19

4 The right-trucanted zeta distribution: $\alpha = 1$ versus free α **27**
4.1 Normalization by constant length in words 28
4.2 Normalization by constant number of different words 28
4.3 Brief discussion 28

5 The range of variation of α: further support for the evolution of α **38**

1. Age ranges of target children

A summary of the age ranges of the target children included in our analyses is shown in Tables 1 for English, Table 2 for German and Table 3 for Dutch and Swedish.

Table 1. Summary of age ranges of target children: English

Language	Corpus	Target child	Age points	Initial age	Final age
English	Lara	Lara	120	21.43	39.83
English	Bloom70	Eric	3	20.23	22.90
English	Bloom70	Peter	20	21.27	37.67
English	Brown	Adam	55	27.13	62.40
English	Brown	Eve	10	18.00	27.00
English	Brown	Sarah	134	27.17	61.20
English	Kuczaj	Abe	207	28.80	60.37
English	Macwhinney	Mark	31	41.87	66.80
English	Macwhinney	Ross	74	16.37	66.80
English	Macwhinney	Unknown1	1	64.50	64.50
English	Macwhinney	Unknown2	1	64.50	64.50
English	Manchester	Anne	35	22.23	33.33
English	Manchester	Aran	33	23.40	34.93
English	Manchester	Becky	34	24.23	35.50
English	Manchester	Carl	33	20.73	32.50
English	Manchester	Dominic	35	22.83	34.53
English	Manchester	Gail	34	23.90	35.40
English	Manchester	Joel	35	23.03	34.37
English	Manchester	John	32	23.50	34.80
English	Manchester	Liz	34	23.30	34.60
English	Manchester	Nicole	33	24.83	36.33
English	Manchester	Ruth	33	23.50	35.70
English	Manchester	Warren	36	22.20	33.67
English	Providence	Alex	51	16.93	41.53
English	Providence	Lily	80	13.07	48.07
English	Providence	Naima	87	11.93	46.33
English	Providence	Violet	51	14.00	47.80
English	Providence	William	44	16.33	40.50
English	Sachs	Naomi	92	14.97	57.10
English	Suppes	Nina	52	23.53	39.70
English	Wells	Abigail	10	17.93	56.00
English	Wells	Benjamin	10	17.70	60.80
English	Wells	Betty	9	18.10	59.07
English	Wells	Darren	10	18.07	58.20
English	Wells	Debbie	16	18.30	47.93
English	Wells	Ellen	10	17.87	57.73
English	Wells	Elspeth	10	18.00	60.10
English	Wells	Frances	10	18.03	58.27
English	Wells	Gary	9	18.00	57.00
English	Wells	Gavin	9	18.70	57.60
English	Wells	Geoffrey	9	18.00	59.73
English	Wells	Gerald	9	18.20	57.17
English	Wells	Harriet	10	18.07	58.10
English	Wells	Iris	9	18.00	56.13
English	Wells	Jack	10	17.87	57.03
English	Wells	Jason	10	18.00	60.63
English	Wells	Jonathan	10	18.17	55.47
English	Wells	Laura	9	18.03	42.07
English	Wells	Lee	8	17.93	41.97
English	Wells	Martin	9	17.87	41.93
English	Wells	Nancy	8	18.07	39.10
English	Wells	Neil	8	18.13	42.03
English	Wells	Neville	9	17.83	41.90
English	Wells	Olivia	9	18.00	41.73
English	Wells	Penny	9	18.30	41.87
English	Wells	Rosie	8	21.63	42.37
English	Wells	Samantha	9	18.20	42.37
English	Wells	Sean	1	18.37	18.37
English	Wells	Sheila	9	21.07	42.83
English	Wells	Simon	9	17.70	41.73
English	Wells	Stella	8	18.27	42.00
English	Wells	Tony	9	17.87	42.27

Ages are given in months. Only the names of the target children employed in our study are shown (target children with less than two time points are excluded). Age points refers to number of different ages before applying the filter that excludes transcripts from 5 years onwards (see Methods in the main article). Initial and final age refer to the age at which the study started and ended, respectively.

Table 2. Summary of age ranges of target children: German

Language	Corpus	Target child	Age points	Initial age	Final age
German	Caroline	Caroline	235	10.03	51.60
German	Leo	Leo	494	23.40	59.17
German	Rigol	Cosima	107	7.10	86.73
German	Rigol	Pauline	97	11.53	95.10
German	Rigol	Sebastian	91	16.07	89.37
German	Szagun	Anna	22	16.17	43.90
German	Szagun	Celina	5	16.03	34.43
German	Szagun	Emely	27	16.20	44.10
German	Szagun	Falko	22	16.00	44.07
German	Szagun	Finng	5	16.17	34.80
German	Szagun	Ina	5	16.00	31.40
German	Szagun	Isabel	5	16.67	34.37
German	Szagun	Jores	3	16.40	25.43
German	Szagun	Konstantin	5	16.27	34.27
German	Szagun	Leo	5	16.33	34.80
German	Szagun	Leon	5	16.17	34.47
German	Szagun	Lisa	22	15.97	43.73
German	Szagun	Luisa	5	16.37	35.00
German	Szagun	Mario	5	16.27	34.60
German	Szagun	Marlou	2	16.30	34.43
German	Szagun	Martin	7	16.40	34.47
German	Szagun	Neele	5	15.33	34.50
German	Szagun	Rahel	22	16.07	43.67
German	Szagun	Sina	5	16.93	34.47
German	Szagun	Sino	5	16.00	34.57
German	Szagun	SRen	22	16.00	44.00

Format as in Table 1.

Table 3. Summary of age ranges of target children: Dutch & Swedish

Language	Corpus	Target child	Age points	Initial age	Final age
Dutch	Groningen	Abel	28	23.00	40.03
Dutch	Groningen	Daan	34	20.70	40.00
Dutch	Groningen	Josse	28	24.23	40.57
Dutch	Groningen	Matthijs	42	22.43	43.07
Dutch	Groningen	Peter	27	17.30	32.73
Dutch	Groningen	Tomas	26	19.17	37.07
Dutch	Schaerlaekens	Arnold	13	22.60	37.23
Dutch	Schaerlaekens	Diederik	13	22.60	37.23
Dutch	Schaerlaekens	Gijs	12	20.97	34.77
Dutch	Schaerlaekens	Joost	12	20.97	34.77
Dutch	Schaerlaekens	Katelijne	12	20.97	34.77
Dutch	Schaerlaekens	Maria	13	22.60	37.23
Dutch	Vankampen	Laura	78	21.13	66.40
Dutch	Vankampen	Sarah	50	18.53	62.43
Swedish	Goteborg	Anton	40	23.27	47.97
Swedish	Goteborg	Harry	40	18.67	47.77
Swedish	Goteborg	Markus	26	15.63	33.97
Swedish	Goteborg	Bel	32	18.30	41.30
Swedish	Goteborg	Tea	34	18.33	47.77

Format as in Table 1.

2. The cut-offs for normalization

The cut-offs for normalization, T^* (by length) and n^* (by observed vocabulary size), were chosen based upon the summary of the raw statistics of T and n in Tables 4 and 5. We focused on the major classes of roles: 'target child', 'father', 'mother' and 'investigator'. $T^* = 500$ and $n^* = 100$ were chosen for being round lower bounds to the smallest mean T and the smallest mean n, respectively, among the major classes of roles at the level of all languages mixed (i.e. the mean T and the mean n of investigators). T^* and n^* were then halved to increase the number of participants and the number of ages considered for each participant, yielding $T^* = 250$ and $n^* = 50$.

Table 4. Analysis of the variation T, the total number of words.

Language	Role class	N	T			
			min	*mean*	*max*	*dev*
All	Target child	101	287.76 ± 296.19	1052.17 ± 780.19	2123.83 ± 1610.10	508.07 ± 395.50
All	Father	24	86.79 ± 169.94	800.64 ± 733.05	2213.25 ± 1688.97	611.28 ± 443.48
All	Investigator	45	180.07 ± 383.62	583.03 ± 784.56	1297.80 ± 1371.12	320.18 ± 314.57
All	Mother	47	870.62 ± 983.34	2317.41 ± 1234.92	4297.34 ± 2097.48	771.72 ± 408.88
All	Other adults	43	42.79 ± 87.31	368.67 ± 489.59	1013.98 ± 1239.08	302.28 ± 356.11
All	Other children	21	43.29 ± 37.89	227.45 ± 199.65	647.76 ± 819.70	209.90 ± 319.53
All	Remainder	8	12.50 ± 6.28	71.25 ± 47.12	352.62 ± 379.02	90.54 ± 88.37
Dutch	Target child	14	146.29 ± 163.33	848.61 ± 371.19	1751.36 ± 804.52	450.69 ± 182.68
Dutch	Father	4	260.50 ± 331.46	907.43 ± 403.43	2103.75 ± 478.71	601.04 ± 171.41
Dutch	Investigator	6	583.67 ± 340.95	1652.11 ± 338.62	3123.67 ± 800.49	639.47 ± 213.75
Dutch	Mother	7	463.00 ± 164.79	1913.59 ± 416.91	3502.86 ± 1205.47	618.22 ± 133.04
Dutch	Other children	1	7.00 ± 0.00	50.32 ± 0.00	259.00 ± 0.00	55.73 ± 0.00
English	Target child	58	287.66 ± 323.98	955.33 ± 909.46	1924.84 ± 1907.47	416.48 ± 433.66
English	Father	13	43.15 ± 61.83	642.19 ± 848.31	1988.23 ± 1919.29	498.28 ± 442.88
English	Investigator	29	149.17 ± 407.44	452.81 ± 804.28	1012.93 ± 1317.90	263.40 ± 325.14
English	Mother	26	1291.08 ± 1124.29	2795.50 ± 1406.85	4674.77 ± 1876.07	752.15 ± 381.11
English	Other adults	31	24.48 ± 27.60	207.30 ± 252.74	677.19 ± 923.52	204.77 ± 295.83
English	Other children	14	42.71 ± 34.54	195.00 ± 162.22	644.36 ± 936.52	217.71 ± 382.30
English	Remainder	7	10.86 ± 4.56	76.28 ± 48.52	396.14 ± 387.20	101.05 ± 89.89
German	Target child	24	386.21 ± 285.92	1413.75 ± 571.53	2858.00 ± 1055.20	773.15 ± 312.20
German	Father	4	7.75 ± 3.20	1004.30 ± 865.96	3154.25 ± 2319.96	926.94 ± 655.79
German	Investigator	10	27.50 ± 29.62	319.19 ± 197.76	1028.40 ± 931.02	293.28 ± 224.40
German	Mother	9	339.33 ± 499.85	1699.18 ± 707.17	4764.89 ± 3106.68	1039.05 ± 589.70
German	Other adults	8	91.00 ± 175.56	739.08 ± 786.29	1805.50 ± 1882.96	512.66 ± 463.17
German	Other children	6	50.67 ± 48.07	332.70 ± 261.74	720.50 ± 608.78	217.36 ± 153.01
German	Remainder	1	24.00 ± 0.00	36.00 ± 0.00	48.00 ± 0.00	16.97 ± 0.00
Swedish	Target child	5	212.60 ± 74.05	1009.80 ± 193.26	1951.00 ± 354.65	458.78 ± 62.32
Swedish	Father	3	149.67 ± 230.65	1073.30 ± 335.43	2079.67 ± 367.70	693.76 ± 320.93
Swedish	Mother	5	211.20 ± 246.66	1509.49 ± 678.05	2605.40 ± 1006.20	607.18 ± 233.20
Swedish	Other adults	4	88.25 ± 121.55	878.39 ± 541.38	2041.00 ± 666.15	637.26 ± 183.78

N is the number of individuals analyzed for a given role class and language category that have at least $m^* = 5$ time points (see Methods for a justification of this lower bound). For each individual, four statistics concerning T are computed: the minimum (*min*), the mean (*mean*), the maximum (*max*) and the standard deviation (*dev*) over all his/her transcripts. The mean plus/minus 1 standard deviation of these four statistics is shown for each role class and language category (when $N = 1$, a standard deviation of 0 is assumed).

Table 5. Analysis of the variation n, the number of different words.

Language	Role class	N	n *min*	*mean*	*max*	*dev*
All	Target child	101	70.41 ± 60.63	221.92 ± 112.05	390.77 ± 192.03	95.07 ± 51.54
All	Father	24	39.71 ± 63.01	200.76 ± 131.87	432.88 ± 222.87	114.29 ± 61.62
All	Investigator	45	60.42 ± 88.71	155.09 ± 126.62	287.53 ± 177.80	66.48 ± 40.89
All	Mother	47	202.04 ± 161.04	440.22 ± 155.20	699.15 ± 223.16	115.10 ± 57.85
All	Other adults	43	22.56 ± 35.84	111.62 ± 109.94	244.49 ± 218.16	70.69 ± 59.97
All	Other children	21	25.71 ± 21.59	79.33 ± 56.79	169.19 ± 114.29	47.68 ± 37.02
All	Remainder	8	7.25 ± 4.56	28.01 ± 10.84	84.25 ± 87.72	21.80 ± 16.84
Dutch	Target child	14	50.57 ± 45.28	192.64 ± 71.01	335.93 ± 124.27	79.72 ± 25.20
Dutch	Father	4	110.75 ± 116.32	263.96 ± 95.38	466.25 ± 88.99	119.50 ± 42.78
Dutch	Investigator	6	197.67 ± 81.32	362.33 ± 43.99	532.50 ± 23.40	85.86 ± 28.82
Dutch	Mother	7	176.29 ± 50.58	425.71 ± 48.36	629.71 ± 86.27	92.79 ± 14.04
Dutch	Other children	1	2.00 ± 0.00	11.32 ± 0.00	88.00 ± 0.00	18.08 ± 0.00
English	Target child	58	68.28 ± 66.85	188.24 ± 111.44	332.90 ± 192.47	73.98 ± 39.40
English	Father	13	26.00 ± 35.49	165.44 ± 141.12	380.77 ± 224.92	94.57 ± 51.17
English	Investigator	29	45.97 ± 78.77	123.24 ± 115.58	240.10 ± 161.76	57.02 ± 39.21
English	Mother	26	264.00 ± 171.24	478.12 ± 173.83	715.46 ± 186.99	100.82 ± 41.14
English	Other adults	31	15.58 ± 15.80	76.96 ± 60.43	188.26 ± 156.76	55.08 ± 49.57
English	Other children	14	26.71 ± 22.05	71.14 ± 33.93	161.14 ± 98.26	46.23 ± 37.69
English	Remainder	7	5.86 ± 2.48	28.80 ± 11.45	92.29 ± 91.51	23.80 ± 17.12
German	Target child	24	86.42 ± 54.72	312.26 ± 91.49	553.17 ± 144.81	154.57 ± 48.44
German	Father	4	6.50 ± 3.51	231.01 ± 170.37	604.75 ± 329.13	171.10 ± 93.75
German	Investigator	10	20.00 ± 21.18	123.13 ± 52.06	278.10 ± 159.66	82.27 ± 45.98
German	Mother	9	105.33 ± 143.15	407.11 ± 142.50	828.00 ± 326.95	182.07 ± 83.50
German	Other adults	8	41.75 ± 70.67	201.77 ± 179.48	392.25 ± 350.27	110.02 ± 83.08
German	Other children	6	27.33 ± 21.96	109.77 ± 87.65	201.50 ± 156.18	56.03 ± 39.02
German	Remainder	1	17.00 ± 0.00	22.50 ± 0.00	28.00 ± 0.00	7.78 ± 0.00
Swedish	Target child	5	73.80 ± 36.69	260.93 ± 45.38	436.20 ± 57.12	97.15 ± 14.20
Swedish	Father	3	48.67 ± 64.38	229.18 ± 64.62	385.00 ± 104.52	117.07 ± 54.51
Swedish	Mother	5	90.00 ± 85.40	323.07 ± 121.22	479.60 ± 146.42	100.08 ± 33.11
Swedish	Other adults	4	38.25 ± 44.99	199.86 ± 114.24	384.75 ± 130.96	113.01 ± 25.28

The same format as in Table 4 is adopted. In our analyses, n is equivalent r_M, one of the parameters of the right-truncated zeta distribution.

3. Normalizations excluded from the main article

3.1. Normalization by prefix: additional tables with lower cut-offs

3.1.1. Dependencies of parameters with age Figures 1 and 2 show the evolution of α with time for cut-offs at $T^* = 250$ and $n^* = 50$, respectively.

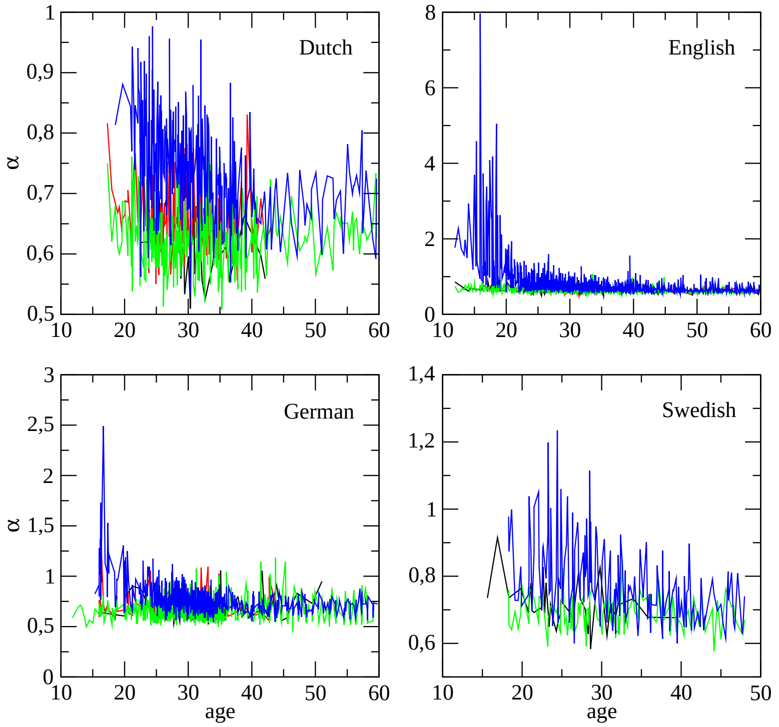

Figure 1. The evolution of the exponent α versus child age (in months): $T^* = 250$. The major classes of roles, i.e. target children (blue), mothers (green), investigators (red) and fathers (black), are shown. Length normalization by prefix with $T^* = 250$ is used. Swedish lacks the class 'investigator'.

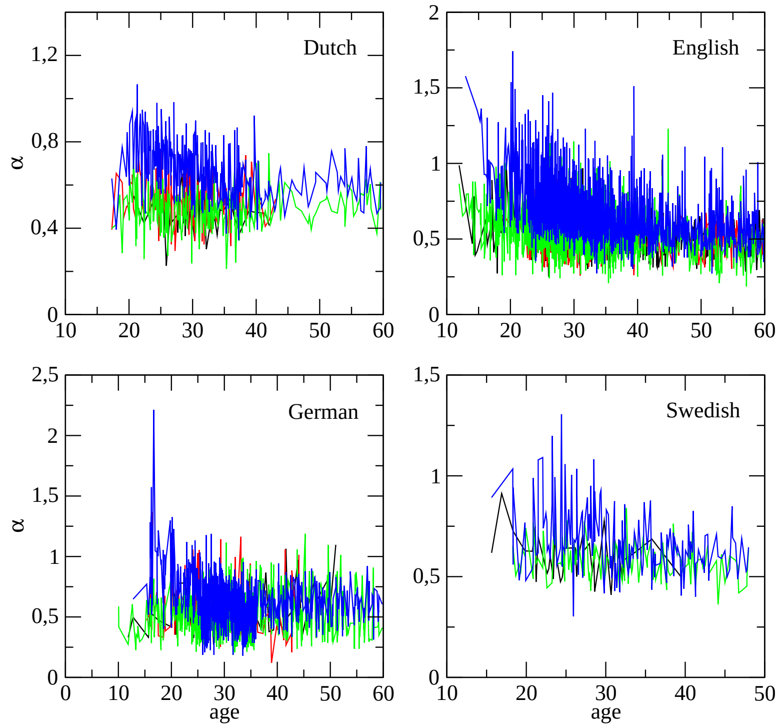

Figure 2. The evolution of the exponent α versus child age (in months): $n^* = 50$. The major classes of roles, i.e. target children (blue), mothers (green), investigators (red) and fathers (black), are shown. Length normalization by prefix with $n^* = 50$ is used. Swedish lacks the class 'investigator'.

Tables 6 and 7 show the results of the analysis of the dependency between α and age for cut-offs at $T^* = 250$ and $n^* = 50$, respectively.

Table 6. The dependency between α and age: length normalization by prefix with $T^* = 250$

Language	Role class	Sign of the dependency			Significance of the correlation			
		N	N_+	N_-	N	N_+^S	N_-^S	$N_?$
All	Target child	90	15 ↓	75 ↑	90	0	44 ↑	46 ↓
All	Father	20	3 ↓	17 ↑	20	0	1	19
All	Investigator	24	5 ↓	19 ↑	24	0	2	22
All	Mother	47	13 ↓	34 ↑	47	1	14 ↑	32 ↓
All	Other adults	13	5	8	13	0	1	12
All	Other children	5	1	4	5	0	0	5
All	Remainder	1	0	1	1	0	0	1
Dutch	Target child	14	4	10	14	0	9 ↑	5 ↓
Dutch	Father	4	0	4	4	0	0	4
Dutch	Investigator	6	1	5	6	0	0	6
Dutch	Mother	7	2	5	7	0	1	6
English	Target child	47	10 ↓	37 ↑	47	0	22 ↑	25 ↓
English	Father	10	2	8	10	0	0	10
English	Investigator	15	3 ↓	12 ↑	15	0	2	13
English	Mother	26	8 ↓	18 ↑	26	0	9 ↑	17 ↓
English	Other adults	6	2	4	6	0	0	6
English	Other children	3	0	3	3	0	0	3
English	Remainder	1	0	1	1	0	0	1
German	Target child	24	1 ↓	23 ↑	24	0	9 ↑	15 ↓
German	Father	3	1	2	3	0	0	3
German	Investigator	3	1	2	3	0	0	3
German	Mother	9	2	7	9	0	3 ↑	6 ↓
German	Other adults	4	1	3	4	0	1	3
German	Other children	2	1	1	2	0	0	2
Swedish	Target child	5	0 ↓	5 ↑	5	0	4 ↑	1 ↓
Swedish	Father	3	0	3	3	0	1	2
Swedish	Mother	5	1	4	5	1	1	3 ↓
Swedish	Other adults	3	2	1	3	0	0	3

Analysis of the correlation between α and age from two perspectives: the sign of the correlation and the significance of the correlations. Four language categories, i.e. All (all languages mixed), Dutch, English, German and Swedish, are considered. N is the number of individuals analyzed for a given role class and language category that had at least $m^* = 5$ different points of time (the minimum number of points needed to show a significant correlation between a parameter and age through a two-sided correlation test at a significance level of 0.05, see the Methods section). This filter was applied for consistency between the analysis of the sign of the dependency and its significance. For each individual, the Spearman rank correlation [1] between age and a certain parameter of the right-truncated distribution was computed. In the analysis of the sign of the correlation, two counts are provided, namely N_+ and N_-, for each role class and language category. N_+ and N_- are, respectively, the number individuals with a positive and negative correlation (regardless of the sign of the correlation). In the analysis of the significance of the correlation, three counts are provided, namely N_+^S, N_-^S and $N_?$, for each role class and language category. N_+^S and N_-^S are the number individuals with a statistically significant positive and negative correlation, respectively. $N_?$ is the number of individuals with a correlation that is not significant. Significance was decided by a two-sided Spearman rank correlation test [1] at a significance level $a = 0.05$. ↑ and ↓ indicate counts that are, respectively, significantly high or significantly low according to a binomial test (see Methods).

Table 7. The dependency between α and age: length normalization by prefix with $n^* = 50$

Language	Role class	Sign of the dependency			Significance of the correlation			
		N	N_+	N_-	N	N_+^S	N_-^S	$N_?$
All	Target child	99	11 ↓	88 ↑	99	0	47 ↑	52 ↓
All	Father	23	10	13	23	0	2	21
All	Investigator	39	15	24	39	0	2	37
All	Mother	47	12 ↓	35 ↑	47	0	8 ↑	39 ↓
All	Other adults	25	10	15	25	0	2	23
All	Other children	11	3	8	11	0	2 ↑	9
All	Remainder	1	0	1	1	0	0	1
Dutch	Target child	14	1 ↓	13 ↑	14	0	8 ↑	6 ↓
Dutch	Father	4	2	2	4	0	0	4
Dutch	Investigator	6	1	5	6	0	0	6
Dutch	Mother	7	3	4	7	0	0	7
English	Target child	56	6 ↓	50 ↑	56	0	26 ↑	30 ↓
English	Father	12	3	9	12	0	2 ↑	10
English	Investigator	24	7 ↓	17 ↑	24	0	1	23
English	Mother	26	6 ↓	20 ↑	26	0	5 ↑	21 ↓
English	Other adults	18	8	10	18	0	1	17
English	Other children	9	2	7	9	0	2 ↑	7
English	Remainder	1	0	1	1	0	0	1
German	Target child	24	4 ↓	20 ↑	24	0	10 ↑	14 ↓
German	Father	4	4	0	4	0	0	4
German	Investigator	9	7	2	9	0	1	8
German	Mother	9	2	7	9	0	2 ↑	7
German	Other adults	4	1	3	4	0	1	3
German	Other children	2	1	1	2	0	0	2
Swedish	Target child	5	0 ↓	5 ↑	5	0	3 ↑	2 ↓
Swedish	Father	3	1	2	3	0	0	3
Swedish	Mother	5	1	4	5	0	1	4
Swedish	Other adults	3	1	2	3	0	0	3

Methods (other than the normalization) and format are the same as in Table 6.

Figure 3 shows evolution of the dependency between r_M and age for a cut-off at $T^* = 250$.

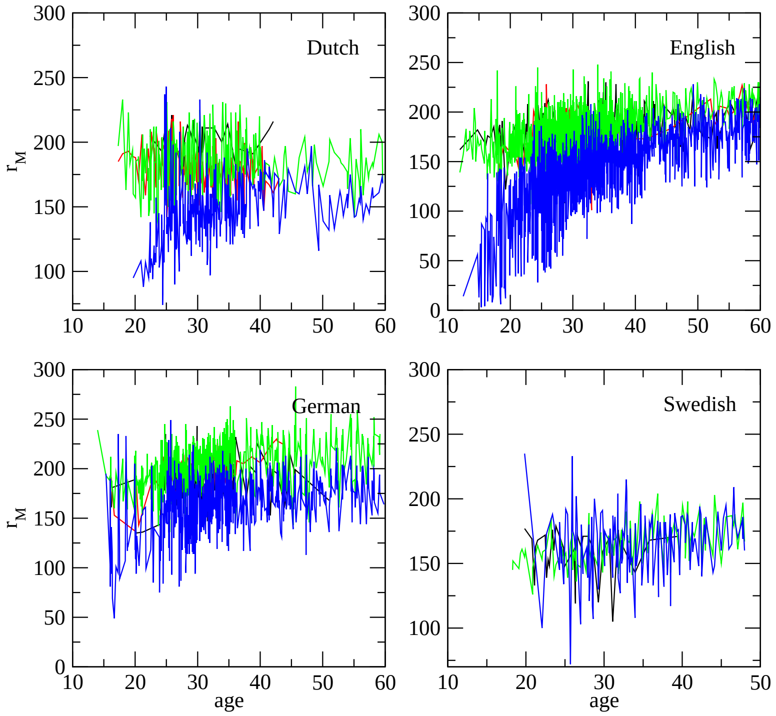

Figure 3. The evolution of the maximum rank r_M versus child age (in months): $T^* = 250$. The major classes of roles, i.e. target children (blue), mothers (green), investigators (red) and fathers (black), are shown. Length normalization by prefix with $T^* = 250$ is used. Swedish lacks the class 'investigator'.

Table 8 shows the results of the analysis of the dependency between r_M and age for this normalization.

Table 8. The dependency between r_M and age: length normalization by prefix with $T^* = 250$

Language	Role class	Sign of the dependency			Significance of the correlation			
		N	N_+	N_-	N	N_+^S	N_-^S	$N_?$
All	Target child	90	82 ↑	8 ↓	90	45 ↑	1	44 ↓
All	Father	20	16 ↑	4 ↓	20	4 ↑	0	16 ↓
All	Investigator	24	16	8	24	3 ↑	1	20 ↓
All	Mother	47	42 ↑	5 ↓	47	20 ↑	1	26 ↓
All	Other adults	13	13 ↑	0 ↓	13	5 ↑	0	8 ↓
All	Other children	5	5 ↑	0 ↓	5	0	0	5
All	Remainder	1	1	0	1	0	0	1
Dutch	Target child	14	13 ↑	1 ↓	14	7 ↑	1	6 ↓
Dutch	Father	4	4	0	4	0	0	4
Dutch	Investigator	6	1	5	6	0	1	5
Dutch	Mother	7	4	3	7	1	1	5 ↓
English	Target child	47	45 ↑	2 ↓	47	24 ↑	0	23 ↓
English	Father	10	8	2	10	3 ↑	0	7 ↓
English	Investigator	15	12 ↑	3 ↓	15	1	0	14
English	Mother	26	25 ↑	1 ↓	26	13 ↑	0	13 ↓
English	Other adults	6	6 ↑	0 ↓	6	1	0	5
English	Other children	3	3	0	3	0	0	3
English	Remainder	1	1	0	1	0	0	1
German	Target child	24	20 ↑	4 ↓	24	11 ↑	0	13 ↓
German	Father	3	3	0	3	0	0	3
German	Investigator	3	3	0	3	2 ↑	0	1 ↓
German	Mother	9	8 ↑	1 ↓	9	3 ↑	0	6 ↓
German	Other adults	4	4	0	4	2 ↑	0	2 ↓
German	Other children	2	2	0	2	0	0	2
Swedish	Target child	5	4	1	5	3 ↑	0	2 ↓
Swedish	Father	3	1	2	3	1	0	2
Swedish	Mother	5	5 ↑	0 ↓	5	3 ↑	0	2 ↓
Swedish	Other adults	3	3	0	3	2 ↑	0	1 ↓

Methods (other than the target parameter) and format are the same as in Table 6.

3.1.2. Dependencies between α and MLU Figures 4 and 5 show the actual dependency between α and MLU for cut-offs at $T^* = 250$ and $n^* = 50$, respectively.

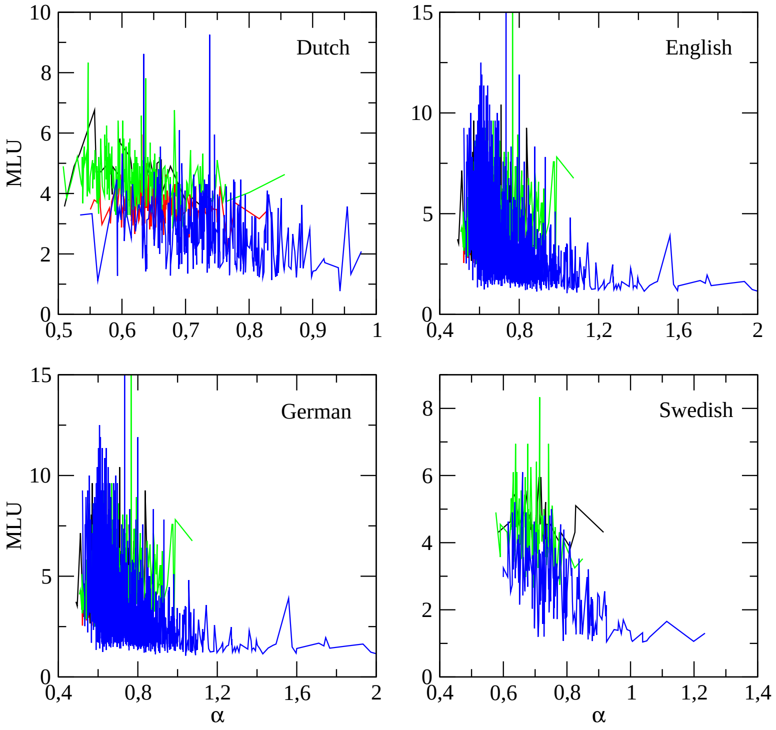

Figure 4. The MLU (in words) versus α: $T^* = 250$. The major classes of roles, i.e. target children (blue), mothers (green), investigators (red) and fathers (black), are shown. Length normalization by prefix with $T^* = 250$ is used. Swedish lacks the class 'investigator'. In order to facilitate the visual inspection of the series, the few points with MLU above 15 or α above 2 are not shown (this concerns English and German).

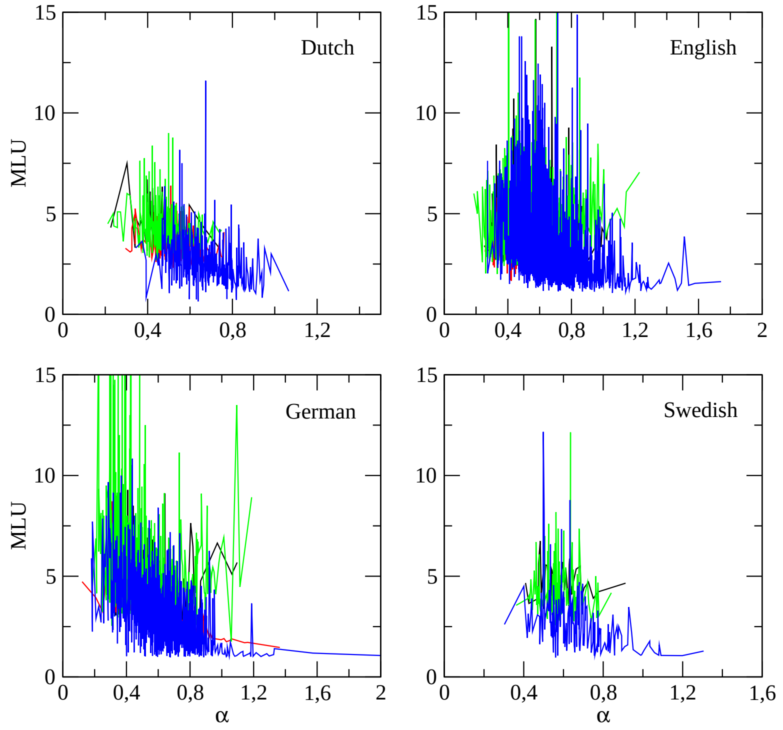

Figure 5. The MLU (in words) versus exponent α: $n^* = 50$. The major classes of roles, i.e. target children (blue), mothers (green), investigators (red) and fathers (black), are shown. Length normalization by prefix with $n^* = 50$ is used. Swedish lacks the class 'investigator'. In order to facilitate the visual inspection of the series, the few points with MLU above 15 or α above 2 are not shown (this concerns English and German).

Tables 9 and 10 show the results of the analysis of the dependency between MLU and age for cut-offs at $T^* = 250$ and $n^* = 50$, respectively.

Table 9. The dependency between α and MLU: length normalization by prefix with $T^* = 250$

Language	Role class	Sign of the dependency			Significance of the correlation			
		N	N_+	N_-	N	N_+^S	N_-^S	$N_?$
All	Target child	90	14 ↓	76 ↑	90	2	40 ↑	48 ↓
All	Father	20	6	14	20	0	5 ↑	15 ↓
All	Investigator	24	8	16	24	0	7 ↑	17 ↓
All	Mother	47	21	26	47	5 ↑	9 ↑	33 ↓
All	Other adults	13	2 ↓	11 ↑	13	0	2 ↑	11
All	Other children	5	1	4	5	0	2 ↑	3 ↓
All	Remainder	1	0	1	1	0	0	1
Dutch	Target child	14	4	10	14	0	8 ↑	6 ↓
Dutch	Father	4	2	2	4	0	1	3
Dutch	Investigator	6	1	5	6	0	2 ↑	4 ↓
Dutch	Mother	7	2	5	7	0	1	6
English	Target child	47	8 ↓	39 ↑	47	2	21 ↑	24 ↓
English	Father	10	3	7	10	0	3 ↑	7 ↓
English	Investigator	15	7	8	15	0	2	13
English	Mother	26	15	11	26	4 ↑	2	20 ↓
English	Other adults	6	1	5	6	0	1	5
English	Other children	3	0	3	3	0	2 ↑	1 ↓
English	Remainder	1	0	1	1	0	0	1
German	Target child	24	2 ↓	22 ↑	24	0	7 ↑	17 ↓
German	Father	3	1	2	3	0	1	2
German	Investigator	3	0	3	3	0	3 ↑	0 ↓
German	Mother	9	2	7	9	1	5 ↑	3 ↓
German	Other adults	4	1	3	4	0	0	4
German	Other children	2	1	1	2	0	0	2
Swedish	Target child	5	0 ↓	5 ↑	5	0	4 ↑	1 ↓
Swedish	Father	3	0	3	3	0	0	3
Swedish	Mother	5	2	3	5	0	1	4
Swedish	Other adults	3	0	3	3	0	1	2

Methods (other than the target variables) and format are the same as in Table 6.

Table 10. The dependency between α and MLU: length normalization by prefix with $n^* = 50$

Language	Role class	Sign of the dependency			Significance of the correlation			
		N	N_+	N_-	N	N_+^S	N_-^S	$N_?$
All	Target child	99	12 ↓	87 ↑	99	1	38 ↑	60 ↓
All	Father	23	11	12	23	0	3 ↑	20
All	Investigator	39	15	24	39	0	6 ↑	33 ↓
All	Mother	47	23	24	47	4 ↑	6 ↑	37 ↓
All	Other adults	25	5 ↓	20 ↑	25	0	1	24
All	Other children	11	3	8	11	0	1	10
All	Remainder	1	0	1	1	0	1 ↑	0 ↓
Dutch	Target child	14	0 ↓	14 ↑	14	0	6 ↑	8 ↓
Dutch	Father	4	2	2	4	0	0	4
Dutch	Investigator	6	2	4	6	0	2 ↑	4 ↓
Dutch	Mother	7	1	6	7	0	0	7
English	Target child	56	8 ↓	48 ↑	56	0	20 ↑	36 ↓
English	Father	12	7	5	12	0	2 ↑	10
English	Investigator	24	12	12	24	0	0	24
English	Mother	26	16	10	26	3 ↑	2	21 ↓
English	Other adults	18	5 ↓	13 ↑	18	0	1	17
English	Other children	9	1 ↓	8 ↑	9	0	1	8
English	Remainder	1	0	1	1	0	1 ↑	0
German	Target child	24	4 ↓	20 ↑	24	1	9 ↑	14 ↓
German	Father	4	1	3	4	0	1	3
German	Investigator	9	1 ↓	8 ↑	9	0	4 ↑	5 ↓
German	Mother	9	3	6	9	1	4 ↑	4 ↓
German	Other adults	4	0	4	4	0	0	4
German	Other children	2	2	0	2	0	0	2
Swedish	Target child	5	0 ↓	5 ↑	5	0	3 ↑	2 ↓
Swedish	Father	3	1	2	3	0	0	3
Swedish	Mother	5	3	2	5	0	0	5
Swedish	Other adults	3	0	3	3	0	0	3

Methods (other than the normalization and the target variables) and format are the same as in Table 6.

3.2. Normalization by random sampling.

3.2.1. Dependency of parameters with age The analysis of the correlation between α and time supports the idea that the behavior of infants and adults differs notably. The analysis of the sign of the correlation between α and age confirms the tendency of α to decrease over time: N_+ is never significantly high while N_- is significantly

large in the majority of target children with the only exception of Swedish, where the number of target children is very small, and also significantly large in investigators and parents depending on the language (Tables 11 and 12 for length normalization; Tables 13 and 14 for observed vocabulary size normalization). If the significance of the correlation between α and age is taken into account, then it turns out that N_+^S is very small (zero in the majority of cases), and never significantly large (Tables 11 and 12 for length normalization; Tables 13 and 14 for observed vocabulary size normalziation). Interestingly, N_-^S is significantly large for all target children (no exception), and the ratio N_-^S/N (where $N = N_+^S + N_-^S + N_?$) in target children is in stark contrast with the that of other classes of roles where N_-^S is significantly large. These results confirm the previous results with normalization by prefix. Furthermore, they suggest that (a) prefix normalization does not omit important information by taking only the beginning of the transcript and (b) the qualitative results do not depend on whether the words selected are consecutive or not.

Table 11. The dependency between α and age: length normalization by random sampling with $T^* = 250$

Language	Role class	Sign of the dependency			Significance of the correlation			
		N	N_+	N_-	N	N_+^S	N_-^S	$N_?$
All	Target child	90	11 ↓	79 ↑	90	1	49 ↑	40 ↓
All	Father	20	6	14	20	0	2	18
All	Investigator	24	7 ↓	17 ↑	24	0	5 ↑	19 ↓
All	Mother	47	13 ↓	34 ↑	47	1	12 ↑	34 ↓
All	Other adults	13	2 ↓	11 ↑	13	0	1	12
All	Other children	5	1	4	5	0	1	4
All	Remainder	1	0	1	1	0	0	1
Dutch	Target child	14	2 ↓	12 ↑	14	0	8 ↑	6 ↓
Dutch	Father	4	1	3	4	0	0	4
Dutch	Investigator	6	2	4	6	0	2 ↑	4 ↓
Dutch	Mother	7	1	6	7	0	1	6
English	Target child	47	8 ↓	39 ↑	47	1	27 ↑	19 ↓
English	Father	10	3	7	10	0	1	9
English	Investigator	15	4	11	15	0	2	13
English	Mother	26	5 ↓	21 ↑	26	1	8 ↑	17 ↓
English	Other adults	6	1	5	6	0	0	6
English	Other children	3	1	2	3	0	1	2
English	Remainder	1	0	1	1	0	0	1
German	Target child	24	0 ↓	24 ↑	24	0	10 ↑	14 ↓
German	Father	3	1	2	3	0	0	3
German	Investigator	3	1	2	3	0	1	2
German	Mother	9	4	5	9	0	2 ↑	7
German	Other adults	4	1	3	4	0	1	3
German	Other children	2	0	2	2	0	0	2
Swedish	Target child	5	1	4	5	0	4 ↑	1 ↓
Swedish	Father	3	1	2	3	0	1	2
Swedish	Mother	5	3	2	5	0	1	4
Swedish	Other adults	3	0	3	3	0	0	3

Methods (other than the normalization) and format are the same as in Table 6.

Table 12. The dependency between α and age: length normalization by random sampling with $T^* = 500$

Language	Role class	Sign of the dependency			Significance of the correlation			
		N	N_+	N_-	N	N_+^S	N_-^S	$N_?$
All	Target child	71	7 ↓	64 ↑	71	2	43 ↑	26 ↓
All	Father	14	4	10	14	0	2 ↑	12
All	Investigator	17	3 ↓	14 ↑	17	0	3 ↑	14
All	Mother	47	14 ↓	33 ↑	47	0	10 ↑	37 ↓
All	Other adults	8	4	4	8	0	2 ↑	6
All	Other children	2	1	1	2	0	0	2
Dutch	Target child	12	2 ↓	10 ↑	12	0	6 ↑	6 ↓
Dutch	Father	2	1	1	2	0	1 ↑	1
Dutch	Investigator	6	1	5	6	0	0	6
Dutch	Mother	7	3	4	7	0	1	6
English	Target child	34	4 ↓	30 ↑	34	2	22 ↑	10 ↓
English	Father	7	1	6	7	0	1	6
English	Investigator	8	2	6	8	0	2 ↑	6
English	Mother	26	4 ↓	22 ↑	26	0	7 ↑	19 ↓
English	Other adults	2	1	1	2	0	0	2
German	Target child	20	0 ↓	20 ↑	20	0	11 ↑	9 ↓
German	Father	3	2	1	3	0	0	3
German	Investigator	3	0	3	3	0	1	2
German	Mother	9	4	5	9	0	1	8
German	Other adults	3	2	1	3	0	1	2
German	Other children	2	1	1	2	0	0	2
Swedish	Target child	5	1	4	5	0	4 ↑	1 ↓
Swedish	Father	2	0	2	2	0	0	2
Swedish	Mother	5	3	2	5	0	1	4
Swedish	Other adults	3	1	2	3	0	1	2

Methods (other than the normalization) and format are the same as in Table 6.

Table 13. The dependency between α and age: length normalization by random sampling with $n^* = 50$

Language	Role class	Sign of the dependency			Significance of the correlation			
		N	N_+	N_-	N	N_+^S	N_-^S	$N_?$
All	Target child	99	9 ↓	90 ↑	99	1	52 ↑	46 ↓
All	Father	23	7 ↓	16 ↑	23	0	1	22
All	Investigator	39	15	24	39	0	2	37
All	Mother	47	9 ↓	38 ↑	47	0	8 ↑	39 ↓
All	Other adults	25	12	13	25	0	4 ↑	21 ↓
All	Other children	11	2 ↓	9 ↑	11	0	1	10
All	Remainder	1	0	1	1	0	0	1
Dutch	Target child	14	2 ↓	12 ↑	14	0	8 ↑	6 ↓
Dutch	Father	4	1	3	4	0	0	4
Dutch	Investigator	6	2	4	6	0	0	6
Dutch	Mother	7	0 ↓	7 ↑	7	0	1	6
English	Target child	56	5 ↓	51 ↑	56	1	29 ↑	26 ↓
English	Father	12	2 ↓	10 ↑	12	0	0	12
English	Investigator	24	10	14	24	0	1	23
English	Mother	26	5 ↓	21 ↑	26	0	5 ↑	21 ↓
English	Other adults	18	9	9	18	0	2	16
English	Other children	9	1 ↓	8 ↑	9	0	1	8
English	Remainder	1	0	1	1	0	0	1
German	Target child	24	1 ↓	23 ↑	24	0	11 ↑	13 ↓
German	Father	4	2	2	4	0	0	4
German	Investigator	9	3	6	9	0	1	8
German	Mother	9	3	6	9	0	1	8
German	Other adults	4	1	3	4	0	1	3
German	Other children	2	1	1	2	0	0	2
Swedish	Target child	5	1	4	5	0	4 ↑	1 ↓
Swedish	Father	3	2	1	3	0	1	2
Swedish	Mother	5	1	4	5	0	1	4
Swedish	Other adults	3	2	1	3	0	1	2

Methods (other than the normalization) and format are the same as in Table 6.

Table 14. The dependency between α and age: length normalization by random sampling with $n^* = 100$

Language	Role class	Sign of the dependency			Significance of the correlation			
		N	N_+	N_-	N	N_+^S	N_-^S	$N_?$
All	Target child	85	12 ↓	73 ↑	85	4	44 ↑	37 ↓
All	Father	19	4 ↓	15 ↑	19	0	2	17
All	Investigator	25	9	16	25	0	5 ↑	20 ↓
All	Mother	47	12 ↓	35 ↑	47	0	14 ↑	33 ↓
All	Other adults	15	3 ↓	12 ↑	15	0	2	13
All	Other children	5	1	4	5	0	0	5
All	Remainder	1	0	1	1	0	0	1
Dutch	Target child	14	1 ↓	13 ↑	14	1	6 ↑	7 ↓
Dutch	Father	4	1	3	4	0	0	4
Dutch	Investigator	6	2	4	6	0	2 ↑	4 ↓
Dutch	Mother	7	2	5	7	0	1	6
English	Target child	46	8 ↓	38 ↑	46	2	23 ↑	21 ↓
English	Father	10	1 ↓	9 ↑	10	0	1	9
English	Investigator	15	6	9	15	0	2	13
English	Mother	26	8 ↓	18 ↑	26	0	11 ↑	15 ↓
English	Other adults	8	2	6	8	0	0	8
English	Other children	3	1	2	3	0	0	3
English	Remainder	1	0	1	1	0	0	1
German	Target child	20	2 ↓	18 ↑	20	0	11 ↑	9 ↓
German	Father	3	2	1	3	0	0	3
German	Investigator	4	1	3	4	0	1	3
German	Mother	9	2	7	9	0	1	8
German	Other adults	4	1	3	4	0	2 ↑	2 ↓
German	Other children	2	0	2	2	0	0	2
Swedish	Target child	5	1	4	5	1	4 ↑	0 ↓
Swedish	Father	2	0	2	2	0	1 ↑	1
Swedish	Mother	5	0 ↓	5 ↑	5	0	1	4
Swedish	Other adults	3	0	3	3	0	0	3

Methods (other than the normalization) and format are the same as in Table 6.

The analysis of the sign of the correlation between r_M and age confirms the tendency of r_M to increase over time: N_- is never significantly high while N_+ is significantly large in the majority of target children with the only exception of Swedish, where the number of target children is very small, and also significantly large in investigators, parents and other adults depending on the language (Tables 15 and 16). If the significance of the correlation between r_M and age is taken into account, then it turns out that N_-^S is very small (zero in the majority of cases), and never significantly large (Tables 15 and

16). Interestingly, N_+^S is significantly large for all target children. With regard to α versus time, the ratio N_+^S/N (where $N = N_+^S + N_-^S + N_?$) is more balanced between target children and the adults where N_+^S is significantly large. These results confirm the previous finding based upon prefix normalization: that the increase of r_M with time does not distinguish children from adults as clearly as α and also confirm that prefix normalization is not omitting vital information.

Table 15. The dependency between r_M and age: length normalization by random sampling with $T^* = 250$

Language	Role class	Sign of the dependency			Significance of the correlation			
		N	N_+	N_-	N	N_+^S	N_-^S	$N_?$
All	Target child	90	80 ↑	10 ↓	90	55 ↑	2	33 ↓
All	Father	20	18 ↑	2 ↓	20	3 ↑	0	17
All	Investigator	24	18 ↑	6 ↓	24	8 ↑	0	16 ↓
All	Mother	47	39 ↑	8 ↓	47	16 ↑	0	31 ↓
All	Other adults	13	13 ↑	0 ↓	13	3 ↑	0	10 ↓
All	Other children	5	5 ↑	0 ↓	5	0	0	5
All	Remainder	1	1	0	1	1 ↑	0	0 ↓
Dutch	Target child	14	13 ↑	1 ↓	14	9 ↑	1	4 ↓
Dutch	Father	4	4	0	4	0	0	4
Dutch	Investigator	6	3	3	6	1	0	5
Dutch	Mother	7	5	2	7	2 ↑	0	5 ↓
English	Target child	47	42 ↑	5 ↓	47	32 ↑	1	14 ↓
English	Father	10	10 ↑	0 ↓	10	1	0	9
English	Investigator	15	12 ↑	3 ↓	15	5 ↑	0	10 ↓
English	Mother	26	21 ↑	5 ↓	26	11 ↑	0	15 ↓
English	Other adults	6	6 ↑	0 ↓	6	1	0	5
English	Other children	3	3	0	3	0	0	3
English	Remainder	1	1	0	1	1 ↑	0	0
German	Target child	24	21 ↑	3 ↓	24	11 ↑	0	13 ↓
German	Father	3	2	1	3	1	0	2
German	Investigator	3	3	0	3	2 ↑	0	1 ↓
German	Mother	9	8 ↑	1 ↓	9	1	0	8
German	Other adults	4	4	0	4	1	0	3
German	Other children	2	2	0	2	0	0	2
Swedish	Target child	5	4	1	5	3 ↑	0	2 ↓
Swedish	Father	3	2	1	3	1	0	2
Swedish	Mother	5	5 ↑	0 ↓	5	2 ↑	0	3 ↓
Swedish	Other adults	3	3	0	3	1	0	2

Methods (other than the normalization and the target parameter) and format are the same as in Table 6.

Table 16. The dependency between r_M and age: length normalization by random sampling with $T^* = 500$

Language	Role class	Sign of the dependency			Significance of the correlation			
		N	N_+	N_-	N	N_+^S	N_-^S	$N_?$
All	Target child	71	65 ↑	6 ↓	71	45 ↑	2	24 ↓
All	Father	14	13 ↑	1 ↓	14	7 ↑	0	7 ↓
All	Investigator	17	11	6	17	4 ↑	0	13 ↓
All	Mother	47	40 ↑	7 ↓	47	19 ↑	0	28 ↓
All	Other adults	8	8 ↑	0 ↓	8	2 ↑	0	6
All	Other children	2	2	0	2	0	0	2
Dutch	Target child	12	11 ↑	1 ↓	12	7 ↑	1	4 ↓
Dutch	Father	2	2	0	2	1 ↑	0	1
Dutch	Investigator	6	3	3	6	0	0	6
Dutch	Mother	7	5	2	7	1	0	6
English	Target child	34	32 ↑	2 ↓	34	25 ↑	0	9 ↓
English	Father	7	7 ↑	0 ↓	7	5 ↑	0	2 ↓
English	Investigator	8	5	3	8	2 ↑	0	6
English	Mother	26	22 ↑	4 ↓	26	12 ↑	0	14 ↓
English	Other adults	2	2	0	2	0	0	2
German	Target child	20	18 ↑	2 ↓	20	11 ↑	0	9 ↓
German	Father	3	2	1	3	1	0	2
German	Investigator	3	3	0	3	2 ↑	0	1 ↓
German	Mother	9	9 ↑	0 ↓	9	3 ↑	0	6 ↓
German	Other adults	3	3	0	3	1	0	2
German	Other children	2	2	0	2	0	0	2
Swedish	Target child	5	4	1	5	2 ↑	1	2 ↓
Swedish	Father	2	2	0	2	0	0	2
Swedish	Mother	5	4	1	5	3 ↑	0	2 ↓
Swedish	Other adults	3	3	0	3	1	0	2

Methods (other than the normalization and the target parameter) and format are the same as in Table 6.

4. The right-trucanted zeta distribution: $\alpha = 1$ versus free α

For each corpus and major class of role (target child, father, investigator and mother), a comparison of the quality of the fit of the two theoretical distributions, i.e. the right-truncated zeta distribution (with two parameters α and r_M) and a right-truncated zeta distribution with only one parameter, i.e. r_M ($\alpha = 1$), is made. The control right-truncated distribution with $\alpha = 1$ was also fitted by maximum likelihood. The maximum likelihood estimator of r_M coincides with n, the maximum rank of the sample.

To see it, consider Eq. 6 of the main text with $\alpha = 1$, $n > 1$ and notice that $H(r_M, 1)$ is a monotonically increasing function of r_M. The quality of the fit was evaluated using Akaike's Information Criterion (AIC), a metric that combines a quantitative measure of the goodness of the fit to the real data with a penalty for the number of parameters used [2]. In our analysis, we adopted a variant that incorporates a correction for small samples which is defined as [3]

$$AIC_k = -2\log(\mathcal{L}) + 2k\frac{T}{T-k-1}, \quad (1)$$

where k is the number of free parameters of the right-truncated zeta distribution ($n = 1$ or $n = 2$ in our case), T is the length of the text sample in words and $\mathcal{L}$ is the log-likelihood as it is defined in the main article. The lower the value of AIC_k of a model with regard to that of alternative models, the better the model.

If no size/length normalization is used, the right-truncated distribution with two parameters gives a better fit in the majority of cases (Table 17).

4.1. Normalization by constant length in words

If fragments of the same T (i.e the same length in words) are considered, the right-truncated distribution of two parameters is better than that of one parameter taking a prefix of length T^* for each time point (see Table 18 for $T^* = 250$ and Table 19 for $T^* = 500$) or taking a random sample of size T^* (see Table 20 for $T^* = 250$ and Table 21 for $T^* = 500$).

4.2. Normalization by constant number of different words

If fragments of the same n (i.e. the same number of different words) are considered, the right-truncated distribution of two parameters is better than that of one parameter taking a prefix of n^* different words for each time point (see Table 22 for $n^* = 50$ and Table 23 for $n^* = 100$) or taking a random sample of n^* different words (see Table 24 for $n^* = 50$ and Table 25 for $n^* = 100$).

4.3. Brief discussion

For all the normalizations considered above and given a language and a class of role, the percentage of cases where the one parameter truncated zeta distribution yields a better fit than the two parameter version is less than 7%. Interestingly, the success of the two parameters drops when no normalization is used, e.g., various combinations of language and role class reach at least 7 in the percentage of times where the one parameter function is better than the two parameter version (recall Table 17). This suggests that normalization improves the adequacy of the truncated zeta distribution with two parameters but this could be simply due to the loss of individuals producing small samples. A sample that that is too small may not contain enough information to discriminate accurately between the one and the two parameter version and may

Table 17. The right-truncated zeta distribution of one parameter versus that of two parameters

Language	Role class	$AIC_1 < AIC_2$	$AIC_1 > AIC_2$	$AIC_1 = AIC_2$
All	Target child	4.24	95.76	0.00
All	Father	3.23	96.77	0.00
All	Investigator	2.71	97.29	0.00
All	Mother	0.63	99.37	0.00
All	Other adults	7.11	92.89	0.00
All	Other children	19.71	80.29	0.00
All	Remainder	20.17	79.83	0.00
Dutch	Target child	2.63	97.37	0.00
Dutch	Father	0.00	100.00	0.00
Dutch	Investigator	0.00	100.00	0.00
Dutch	Mother	0.00	100.00	0.00
Dutch	Other children	56.00	44.00	0.00
English	Target child	3.54	96.46	0.00
English	Father	3.34	96.66	0.00
English	Investigator	2.12	97.88	0.00
English	Mother	0.19	99.81	0.00
English	Other adults	11.06	88.94	0.00
English	Other children	14.79	85.21	0.00
English	Remainder	20.51	79.49	0.00
German	Target child	5.60	94.40	0.00
German	Father	4.21	95.79	0.00
German	Investigator	7.49	92.51	0.00
German	Mother	1.72	98.28	0.00
German	Other adults	1.89	98.11	0.00
German	Other children	17.76	82.24	0.00
German	Remainder	0.00	100.00	0.00
Swedish	Target child	6.40	93.60	0.00
Swedish	Father	0.00	100.00	0.00
Swedish	Mother	0.00	100.00	0.00
Swedish	Other adults	1.64	98.36	0.00

AIC_1 and AIC_2 are, respectively, the corrected Akaike information criterion for the right-truncated zeta distribution with two parameters (α and r_M) and that of one parameter ($\alpha = 1$ and free r_M). For each language category and role class, the percentage of times (over all the available individual - age pairs where the fit can be performed) that $AIC_1 < AIC_2$, $AIC_1 > AIC_2$ and $AIC_1 = AIC_2$ are shown.

not reach the cut-off imposed for normalization. In fact, various classes of roles do not survive normalization (they are present in Table 17 but disappeared in normalization

Table 18. The right-truncated zeta distribution of one parameter versus that of two parameters: prefixes of constant $T^* = 250$

Language	Role class	$AIC_1 < AIC_2$	$AIC_1 > AIC_2$	$AIC_1 = AIC_2$
All	Target child	3.85	96.15	0.00
All	Father	0.00	100.00	0.00
All	Investigator	0.82	99.18	0.00
All	Mother	0.35	99.65	0.00
All	Other adults	0.00	100.00	0.00
All	Other children	2.08	97.92	0.00
All	Remainder	0.00	100.00	0.00
Dutch	Target child	1.17	98.83	0.00
Dutch	Father	0.00	100.00	0.00
Dutch	Investigator	0.00	100.00	0.00
Dutch	Mother	0.00	100.00	0.00
English	Target child	4.43	95.57	0.00
English	Father	0.00	100.00	0.00
English	Investigator	0.00	100.00	0.00
English	Mother	0.29	99.71	0.00
English	Other adults	0.00	100.00	0.00
English	Other children	3.23	96.77	0.00
English	Remainder	0.00	100.00	0.00
German	Target child	3.42	96.58	0.00
German	Father	0.00	100.00	0.00
German	Investigator	4.49	95.51	0.00
German	Mother	0.65	99.35	0.00
German	Other adults	0.00	100.00	0.00
German	Other children	1.54	98.46	0.00
Swedish	Target child	5.99	94.01	0.00
Swedish	Father	0.00	100.00	0.00
Swedish	Mother	0.00	100.00	0.00
Swedish	Other adults	0.00	100.00	0.00

Comparison of AICs for prefixes of the same length T in words ($T^* = 250$). The same format as in Table 17 is adopted.

tables).

Table 19. The right-truncated zeta distribution of one parameter versus that of two parameters: prefixes of constant $T^* = 500$

Language	Role class	$AIC_1 < AIC_2$	$AIC_1 > AIC_2$	$AIC_1 = AIC_2$
All	Target child	3.16	96.84	0.00
All	Father	1.01	98.99	0.00
All	Investigator	0.31	99.69	0.00
All	Mother	0.17	99.83	0.00
All	Other adults	0.00	100.00	0.00
All	Other children	0.00	100.00	0.00
All	Remainder	0.00	100.00	0.00
Dutch	Target child	0.35	99.65	0.00
Dutch	Father	0.00	100.00	0.00
Dutch	Investigator	0.00	100.00	0.00
Dutch	Mother	0.00	100.00	0.00
English	Target child	3.47	96.53	0.00
English	Father	0.00	100.00	0.00
English	Investigator	0.00	100.00	0.00
English	Mother	0.25	99.75	0.00
English	Other adults	0.00	100.00	0.00
English	Other children	0.00	100.00	0.00
English	Remainder	0.00	100.00	0.00
German	Target child	3.74	96.26	0.00
German	Father	2.92	97.08	0.00
German	Investigator	2.00	98.00	0.00
German	Mother	0.14	99.86	0.00
German	Other adults	0.00	100.00	0.00
German	Other children	0.00	100.00	0.00
Swedish	Target child	2.80	97.20	0.00
Swedish	Father	0.00	100.00	0.00
Swedish	Mother	0.00	100.00	0.00
Swedish	Other adults	0.00	100.00	0.00

Comparison of AICs for prefixes of the same length T in words ($T^* = 500$). The same format as in Table 17 is adopted.

Table 20. The right-truncated zeta distribution of one parameter versus that of two parameters: random samples of constant $T^* = 250$

Language	Role class	$AIC_1 < AIC_2$	$AIC_1 > AIC_2$	$AIC_1 = AIC_2$
All	Target child	2.36	97.64	0.00
All	Father	0.00	100.00	0.00
All	Investigator	0.41	99.59	0.00
All	Mother	0.12	99.88	0.00
All	Other adults	0.00	100.00	0.00
All	Other children	1.04	98.96	0.00
All	Remainder	0.00	100.00	0.00
Dutch	Target child	0.00	100.00	0.00
Dutch	Father	0.00	100.00	0.00
Dutch	Investigator	0.00	100.00	0.00
Dutch	Mother	0.00	100.00	0.00
English	Target child	3.05	96.95	0.00
English	Father	0.00	100.00	0.00
English	Investigator	0.00	100.00	0.00
English	Mother	0.07	99.93	0.00
English	Other adults	0.00	100.00	0.00
English	Other children	3.23	96.77	0.00
English	Remainder	0.00	100.00	0.00
German	Target child	1.89	98.11	0.00
German	Father	0.00	100.00	0.00
German	Investigator	2.25	97.75	0.00
German	Mother	0.26	99.74	0.00
German	Other adults	0.00	100.00	0.00
German	Other children	0.00	100.00	0.00
Swedish	Target child	2.99	97.01	0.00
Swedish	Father	0.00	100.00	0.00
Swedish	Mother	0.00	100.00	0.00
Swedish	Other adults	0.00	100.00	0.00

Comparison of AICs for random samples of the same length T in words ($T^* = 250$). The same format as in Table 17 is adopted.

Table 21. The right-truncated zeta distribution of one parameter versus that of two parameters: random samples of constant $T^* = 500$

Language	Role class	$AIC_1 < AIC_2$	$AIC_1 > AIC_2$	$AIC_1 = AIC_2$
All	Target child	2.38	97.62	0.00
All	Father	1.01	98.99	0.00
All	Investigator	0.31	99.69	0.00
All	Mother	0.00	100.00	0.00
All	Other adults	0.00	100.00	0.00
All	Other children	0.00	100.00	0.00
All	Remainder	0.00	100.00	0.00
Dutch	Target child	0.35	99.65	0.00
Dutch	Father	0.00	100.00	0.00
Dutch	Investigator	0.00	100.00	0.00
Dutch	Mother	0.00	100.00	0.00
English	Target child	3.18	96.82	0.00
English	Father	0.00	100.00	0.00
English	Investigator	0.00	100.00	0.00
English	Mother	0.00	100.00	0.00
English	Other adults	0.00	100.00	0.00
English	Other children	0.00	100.00	0.00
English	Remainder	0.00	100.00	0.00
German	Target child	1.74	98.26	0.00
German	Father	2.92	97.08	0.00
German	Investigator	2.00	98.00	0.00
German	Mother	0.00	100.00	0.00
German	Other adults	0.00	100.00	0.00
German	Other children	0.00	100.00	0.00
Swedish	Target child	2.10	97.90	0.00
Swedish	Father	0.00	100.00	0.00
Swedish	Mother	0.00	100.00	0.00
Swedish	Other adults	0.00	100.00	0.00

Comparison of AICs for random samples of the same length T in words ($T^* = 500$). The same format as in Table 17 is adopted.

Table 22. The right-truncated zeta distribution of one parameter versus that of two parameters: prefixes of constant $n^* = 50$

Language	Role class	$AIC_1 < AIC_2$	$AIC_1 > AIC_2$	$AIC_1 = AIC_2$
All	Target child	4.61	95.39	0.00
All	Father	1.05	98.95	0.00
All	Investigator	1.12	98.88	0.00
All	Mother	0.95	99.05	0.00
All	Other adults	0.80	99.20	0.00
All	Other children	2.16	97.84	0.00
All	Remainder	4.76	95.24	0.00
Dutch	Target child	1.91	98.09	0.00
Dutch	Father	0.00	100.00	0.00
Dutch	Investigator	0.00	100.00	0.00
Dutch	Mother	0.00	100.00	0.00
English	Target child	6.04	93.96	0.00
English	Father	1.17	98.83	0.00
English	Investigator	0.22	99.78	0.00
English	Mother	1.06	98.94	0.00
English	Other adults	0.93	99.07	0.00
English	Other children	4.35	95.65	0.00
English	Remainder	4.76	95.24	0.00
German	Target child	3.29	96.71	0.00
German	Father	1.18	98.82	0.00
German	Investigator	5.26	94.74	0.00
German	Mother	1.22	98.78	0.00
German	Other adults	0.85	99.15	0.00
German	Other children	0.00	100.00	0.00
Swedish	Target child	4.12	95.88	0.00
Swedish	Father	0.00	100.00	0.00
Swedish	Mother	0.00	100.00	0.00
Swedish	Other adults	0.00	100.00	0.00

Comparison of AICs for prefixes of the same number n of different words ($n^* = 50$). The same format as in Table 17 is adopted.

Table 23. The right-truncated zeta distribution of one parameter versus that of two parameters: prefixes of constant $n^* = 100$

Language	Role class	$AIC_1 < AIC_2$	$AIC_1 > AIC_2$	$AIC_1 = AIC_2$
All	Target child	4.61	95.39	0.00
All	Father	1.05	98.95	0.00
All	Investigator	1.12	98.88	0.00
All	Mother	0.95	99.05	0.00
All	Other adults	0.80	99.20	0.00
All	Other children	2.16	97.84	0.00
All	Remainder	4.76	95.24	0.00
Dutch	Target child	1.91	98.09	0.00
Dutch	Father	0.00	100.00	0.00
Dutch	Investigator	0.00	100.00	0.00
Dutch	Mother	0.00	100.00	0.00
English	Target child	6.04	93.96	0.00
English	Father	1.17	98.83	0.00
English	Investigator	0.22	99.78	0.00
English	Mother	1.06	98.94	0.00
English	Other adults	0.93	99.07	0.00
English	Other children	4.35	95.65	0.00
English	Remainder	4.76	95.24	0.00
German	Target child	3.29	96.71	0.00
German	Father	1.18	98.82	0.00
German	Investigator	5.26	94.74	0.00
German	Mother	1.22	98.78	0.00
German	Other adults	0.85	99.15	0.00
German	Other children	0.00	100.00	0.00
Swedish	Target child	4.12	95.88	0.00
Swedish	Father	0.00	100.00	0.00
Swedish	Mother	0.00	100.00	0.00
Swedish	Other adults	0.00	100.00	0.00

Comparison of AICs for prefixes of the same number n of different words ($n^* = 100$). The same format as in Table 17 is adopted.

Table 24. The right-truncated zeta distribution of one parameter versus that of two parameters: random samples of constant $n^* = 50$

Language	Role class	$AIC_1 < AIC_2$	$AIC_1 > AIC_2$	$AIC_1 = AIC_2$
All	Target child	2.24	97.76	0.00
All	Father	0.00	100.00	0.00
All	Investigator	1.00	99.00	0.00
All	Mother	0.11	99.89	0.00
All	Other adults	0.00	100.00	0.00
All	Other children	0.00	100.00	0.00
All	Remainder	0.00	100.00	0.00
Dutch	Target child	1.09	98.91	0.00
Dutch	Father	0.00	100.00	0.00
Dutch	Investigator	0.00	100.00	0.00
Dutch	Mother	0.00	100.00	0.00
English	Target child	2.36	97.64	0.00
English	Father	0.00	100.00	0.00
English	Investigator	0.22	99.78	0.00
English	Mother	0.07	99.93	0.00
English	Other adults	0.00	100.00	0.00
English	Other children	0.00	100.00	0.00
English	Remainder	0.00	100.00	0.00
German	Target child	2.36	97.64	0.00
German	Father	0.00	100.00	0.00
German	Investigator	4.61	95.39	0.00
German	Mother	0.24	99.76	0.00
German	Other adults	0.00	100.00	0.00
German	Other children	0.00	100.00	0.00
Swedish	Target child	2.94	97.06	0.00
Swedish	Father	0.00	100.00	0.00
Swedish	Mother	0.00	100.00	0.00
Swedish	Other adults	0.00	100.00	0.00

Comparison of AICs for random samples of the same number n of different words ($n^* = 50$). The same format as in Table 17 is adopted.

Table 25. The right-truncated zeta distribution of one parameter versus that of two parameters: random samples of constant $n^* = 100$

Language	Role class	$AIC_1 < AIC_2$	$AIC_1 > AIC_2$	$AIC_1 = AIC_2$
All	Target child	1.60	98.40	0.00
All	Father	0.17	99.83	0.00
All	Investigator	0.55	99.45	0.00
All	Mother	0.08	99.92	0.00
All	Other adults	0.00	100.00	0.00
All	Other children	0.00	100.00	0.00
All	Remainder	0.00	100.00	0.00
Dutch	Target child	0.00	100.00	0.00
Dutch	Father	0.00	100.00	0.00
Dutch	Investigator	0.00	100.00	0.00
Dutch	Mother	0.00	100.00	0.00
English	Target child	1.96	98.04	0.00
English	Father	0.00	100.00	0.00
English	Investigator	0.00	100.00	0.00
English	Mother	0.00	100.00	0.00
English	Other adults	0.00	100.00	0.00
English	Other children	0.00	100.00	0.00
English	Remainder	0.00	100.00	0.00
German	Target child	1.42	98.58	0.00
German	Father	0.66	99.34	0.00
German	Investigator	3.06	96.94	0.00
German	Mother	0.25	99.75	0.00
German	Other adults	0.00	100.00	0.00
German	Other children	0.00	100.00	0.00
Swedish	Target child	2.50	97.50	0.00
Swedish	Father	0.00	100.00	0.00
Swedish	Mother	0.00	100.00	0.00
Swedish	Other adults	0.00	100.00	0.00

Comparison of AICs for random samples the same number n of different words ($n^* = 100$). The same format as in Table 17 is adopted.

5. The range of variation of α: further support for the evolution of α

Tables 26 and 27 show the range of variation of α for normalization by prefix at lower cut-offs than those considered in the main article, $T^* = 250$ and $n^* = 50$, respectively.

Table 26. Analysis of the variation the value of the exponent α: $T^* = 250$

Language	Role class	N	α			
			min	*mean*	*max*	*dev*
All	Target child	97	0.63 ± 0.06	0.78 ± 0.13	1.16 ± 0.93	0.13 ± 0.17
All	Father	21	0.58 ± 0.06	0.67 ± 0.07	0.81 ± 0.11	0.07 ± 0.03
All	Investigator	35	0.60 ± 0.05	0.66 ± 0.05	0.76 ± 0.14	0.05 ± 0.03
All	Mother	47	0.55 ± 0.04	0.66 ± 0.05	0.82 ± 0.12	0.06 ± 0.03
All	Other adults	24	0.62 ± 0.07	0.70 ± 0.05	0.78 ± 0.08	0.06 ± 0.04
All	Other children	11	0.63 ± 0.05	0.70 ± 0.06	0.80 ± 0.12	0.07 ± 0.04
All	Remainder	2	0.72 ± 0.19	0.91 ± 0.40	1.14 ± 0.57	0.28 ± 0.31
Dutch	Target child	14	0.64 ± 0.07	0.76 ± 0.04	0.90 ± 0.05	0.07 ± 0.02
Dutch	Father	4	0.55 ± 0.03	0.62 ± 0.03	0.67 ± 0.05	0.04 ± 0.02
Dutch	Investigator	6	0.57 ± 0.02	0.66 ± 0.02	0.77 ± 0.05	0.05 ± 0.01
Dutch	Mother	7	0.53 ± 0.02	0.61 ± 0.03	0.73 ± 0.07	0.04 ± 0.01
English	Target child	54	0.61 ± 0.05	0.76 ± 0.14	1.23 ± 1.22	0.14 ± 0.20
English	Father	11	0.56 ± 0.04	0.66 ± 0.04	0.81 ± 0.08	0.07 ± 0.02
English	Investigator	21	0.59 ± 0.05	0.65 ± 0.03	0.72 ± 0.04	0.04 ± 0.02
English	Mother	26	0.55 ± 0.02	0.65 ± 0.03	0.82 ± 0.10	0.06 ± 0.02
English	Other adults	15	0.61 ± 0.05	0.69 ± 0.05	0.77 ± 0.09	0.07 ± 0.04
English	Other children	8	0.63 ± 0.05	0.70 ± 0.07	0.78 ± 0.12	0.07 ± 0.05
English	Remainder	2	0.72 ± 0.19	0.91 ± 0.40	1.14 ± 0.57	0.28 ± 0.31
German	Target child	24	0.66 ± 0.08	0.84 ± 0.12	1.18 ± 0.35	0.17 ± 0.14
German	Father	3	0.62 ± 0.10	0.76 ± 0.13	0.94 ± 0.11	0.10 ± 0.03
German	Investigator	8	0.62 ± 0.05	0.69 ± 0.09	0.87 ± 0.25	0.08 ± 0.06
German	Mother	9	0.55 ± 0.05	0.69 ± 0.07	0.90 ± 0.15	0.09 ± 0.04
German	Other adults	5	0.60 ± 0.10	0.68 ± 0.07	0.78 ± 0.06	0.05 ± 0.03
German	Other children	3	0.61 ± 0.03	0.70 ± 0.01	0.85 ± 0.13	0.06 ± 0.02
Swedish	Target child	5	0.62 ± 0.03	0.78 ± 0.05	1.04 ± 0.13	0.10 ± 0.02
Swedish	Father	3	0.66 ± 0.07	0.72 ± 0.02	0.83 ± 0.08	0.05 ± 0.03
Swedish	Mother	5	0.61 ± 0.02	0.69 ± 0.01	0.79 ± 0.04	0.05 ± 0.01
Swedish	Other adults	4	0.68 ± 0.05	0.74 ± 0.02	0.82 ± 0.03	0.04 ± 0.01

Length normalization by prefix with $T^* = 250$ is used. N is the number of individuals analyzed for a given role class and language category that have at least five time points (for consistency with the minimum number of points of the correlation analysis; see Methods). For each individual, four statistics concerning α are computed: the minimum (*min*), the mean (*mean*), the maximum (*max*) and the standard deviation (*dev*) are calculated over all his/her transcripts. The mean plus/minus 1 standard deviation of these four statistics is shown for each role class and language category (when $N = 1$, a standard deviation of 0 is assumed).

.

Table 27. Analysis of the variation the value of the exponent α: $n^* = 50$

Language	Role class	N	α			
			min	*mean*	*max*	*dev*
All	Target child	101	0.47 ± 0.11	0.70 ± 0.11	1.01 ± 0.25	0.15 ± 0.08
All	Father	23	0.37 ± 0.10	0.56 ± 0.09	0.81 ± 0.15	0.12 ± 0.04
All	Investigator	44	0.36 ± 0.08	0.53 ± 0.07	0.74 ± 0.19	0.11 ± 0.05
All	Mother	47	0.32 ± 0.08	0.54 ± 0.06	0.84 ± 0.17	0.11 ± 0.03
All	Other adults	35	0.44 ± 0.13	0.58 ± 0.10	0.74 ± 0.13	0.10 ± 0.05
All	Other children	13	0.44 ± 0.08	0.60 ± 0.10	0.82 ± 0.20	0.12 ± 0.04
All	Remainder	2	0.60 ± 0.33	0.83 ± 0.42	1.17 ± 0.54	0.24 ± 0.19
Dutch	Target child	14	0.48 ± 0.09	0.69 ± 0.05	0.91 ± 0.07	0.11 ± 0.02
Dutch	Father	4	0.34 ± 0.10	0.47 ± 0.06	0.61 ± 0.11	0.09 ± 0.04
Dutch	Investigator	6	0.34 ± 0.04	0.52 ± 0.03	0.71 ± 0.06	0.09 ± 0.02
Dutch	Mother	7	0.28 ± 0.06	0.47 ± 0.04	0.65 ± 0.06	0.08 ± 0.01
English	Target child	58	0.46 ± 0.09	0.68 ± 0.10	0.97 ± 0.24	0.14 ± 0.05
English	Father	12	0.34 ± 0.07	0.57 ± 0.07	0.85 ± 0.13	0.13 ± 0.04
English	Investigator	28	0.36 ± 0.07	0.51 ± 0.06	0.70 ± 0.13	0.09 ± 0.02
English	Mother	26	0.32 ± 0.07	0.55 ± 0.06	0.88 ± 0.16	0.12 ± 0.03
English	Other adults	26	0.44 ± 0.12	0.58 ± 0.09	0.74 ± 0.12	0.11 ± 0.05
English	Other children	10	0.46 ± 0.07	0.60 ± 0.11	0.78 ± 0.19	0.12 ± 0.04
English	Remainder	2	0.60 ± 0.33	0.83 ± 0.42	1.17 ± 0.54	0.24 ± 0.19
German	Target child	24	0.50 ± 0.16	0.76 ± 0.13	1.14 ± 0.31	0.20 ± 0.12
German	Father	4	0.41 ± 0.16	0.59 ± 0.16	0.87 ± 0.18	0.13 ± 0.04
German	Investigator	10	0.34 ± 0.11	0.56 ± 0.09	0.89 ± 0.30	0.16 ± 0.09
German	Mother	9	0.30 ± 0.09	0.53 ± 0.07	0.88 ± 0.22	0.13 ± 0.05
German	Other adults	5	0.38 ± 0.17	0.56 ± 0.14	0.78 ± 0.20	0.11 ± 0.01
German	Other children	3	0.39 ± 0.08	0.59 ± 0.01	0.94 ± 0.21	0.14 ± 0.04
Swedish	Target child	5	0.43 ± 0.08	0.67 ± 0.08	1.09 ± 0.13	0.14 ± 0.03
Swedish	Father	3	0.47 ± 0.06	0.61 ± 0.03	0.79 ± 0.11	0.09 ± 0.02
Swedish	Mother	5	0.43 ± 0.06	0.58 ± 0.02	0.78 ± 0.03	0.08 ± 0.01
Swedish	Other adults	4	0.52 ± 0.08	0.64 ± 0.02	0.75 ± 0.05	0.07 ± 0.03

Observed vocabulary size normalization by prefix with $n^* = 50$ is used. The remainder of the methods and the format are the same as in Table 26.

References

[1] W. J. Conover. *Practical nonparametric statistics.* Wiley, New York, 1999. 3rd edition.

[2] H. Akaike. Information theory as an extension of the maximum likelihood principle. In B. N. Petrov and F. Csaki, editors, *Second International Symposium on Information Theory*, pages 267–281. Akademiai Kiado, Budapest, 1973.

[3] N. Sugiura. Further analysis of the data by Akaike's information criterion and the finite corrections. *Communication and Statistics - Theory and Methods*, 7:13–26, 1978.
